# A review and analysis of key biomarkers in Alzheimer’s disease

**DOI:** 10.3389/fnins.2024.1358998

**Published:** 2024-02-20

**Authors:** Zhihao Zhang, Xiangtao Liu, Suixia Zhang, Zhixin Song, Ke Lu, Wenzhong Yang

**Affiliations:** ^1^School of Computer Science and Technology, Xinjiang University, Ürümqi, China; ^2^College of Medical Engineering and Technology, Xinjiang Medical University, Ürümqi, China; ^3^College of Biomedical Engineering and Instrument Science, Zhejiang University, Hangzhou, China; ^4^State Key Laboratory of Pathogenesis, Prevention, Treatment of Central Asian High Incidence Diseases, First Affiliated Hospital of Xinjiang Medical University, Ürümqi, China

**Keywords:** review, AD biomarker, GTEx, GEO, tissue-specific

## Abstract

Alzheimer’s disease (AD) is a progressive neurodegenerative disorder that affects over 50 million elderly individuals worldwide. Although the pathogenesis of AD is not fully understood, based on current research, researchers are able to identify potential biomarker genes and proteins that may serve as effective targets against AD. This article aims to present a comprehensive overview of recent advances in AD biomarker identification, with highlights on the use of various algorithms, the exploration of relevant biological processes, and the investigation of shared biomarkers with co-occurring diseases. Additionally, this article includes a statistical analysis of key genes reported in the research literature, and identifies the intersection with AD-related gene sets from databases such as AlzGen, GeneCard, and DisGeNet. For these gene sets, besides enrichment analysis, protein–protein interaction (PPI) networks utilized to identify central genes among the overlapping genes. Enrichment analysis, protein interaction network analysis, and tissue-specific connectedness analysis based on GTEx database performed on multiple groups of overlapping genes. Our work has laid the foundation for a better understanding of the molecular mechanisms of AD and more accurate identification of key AD markers.

## 1 Introduction

Alzheimer’s disease (AD) is a progressive neurodegenerative disorder that affects over 50 million elderly individuals worldwide (Alzheimer’s Statistics, 2019). Late-onset Alzheimer’s Disease (LOAD) accounts for more than 97% of all AD cases and typically occurs after the age of 65. Along with the global trend of population aging, the incidence rate of AD has risen substantially. The World Health Organization’s “Global Health Estimates 2019” report (World Health Statistics, 2019^1^) revealed that Alzheimer’s disease and other forms of dementia have entered the top ten leading causes of death over the past 20 years. According to the 2021 special report “Race, Ethnicity, and Alzheimer’s Disease in America” by Alzheimer’s Association (Alzheimer’s Association, 2023^2^), more than 6 million Americans suffer from AD, with the number of deaths surpassing the combined total deaths from breast and prostate cancers. According to a nationwide cross-sectional study conducted in 2020 (Chou et al., 2020), there are 15.07 million cases of dementia among the population aged 60 and above in China, including 9.83 million cases of AD, 3.92 million cases of vascular dementia, and 1.32 million cases of other types of dementia. During the COVID-19 pandemic, the number of deaths among AD and other dementia patients increased by 16% in the U.S. The Centers for Disease Control and Prevention in the United States reported that the number of elderly dementia patients aged 65 and above doubles every five years (Centers for Disease Control and Prevention, 2020^3^).

Individuals with AD generally experience a range of phenotypic changes, including memory loss, cognitive decline, and impaired executive function (Alzheimer’s Statistics, 2019). The major symptoms include memory decline, impaired language abilities, diminished judgment, abnormal behaviors, and emotions, as well as severe loss of daily life skills (Hane et al., 2017). AD is classified as familial and sporadic. The dominant familial or autosomal presentation represents 1–5% of the total number of cases. It is categorized as early onset (EOAD; <65 years of age) and presents genetic mutations in presenilin 1 (PSEN1), presenilin 2 (PSEN2), or the Amyloid precursor protein (APP). Sporadic AD represents 95% of the cases and is categorized as late-onset (LOAD), occurring in patients older than 65 years of age (Andrade-Guerrero et al., 2023). Specific mechanisms and causes of AD remain unclear, but it is believed to be related to genetics, brain injuries, and environmental factors. Two monoclonal antibody drugs aducanumab (Knopman et al., 2021; Haeberlein et al., 2022) and lecanemab (van Dyck et al., 2023) have been approved for Alzheimer’s disease treatment. It is the first new therapy approved for the treatment of this disease in nearly 20 years and the first therapy to modify the disease. Patients require long-term treatment and caregiving management, which poses significant challenges and exerts a profound impact on their families, while also resulting in substantial societal costs. The hallmark features of this disease are the formation of amyloid-beta plaques and the tangles of tau protein fibers (Calabrese et al., 2008). These abnormal structures impair neuronal cells, brain volume, and cognitive abilities, leading to compromised and lost connections between nerve cells (Sampath et al., 2017). Despite the incomplete knowledge of exact pathogenetic mechanisms, measurement of α-beta and p-tau protein levels in cerebrospinal fluid (CSF), can aid in AD diagnosis (Zhu et al., 2023a). However, the invasive nature of lumbar puncture and the high cost of PET scans have limited the application of these methods. With the advancement of high-throughput sequencing and microarray technologies, bioinformatics has been increasingly utilized to analyze genetic alterations in the nervous system. At present, many biomarkers and targets are identified primarily through computational methods, aiming to minimize the substantial investment required for drug development.

**Figure 1:**
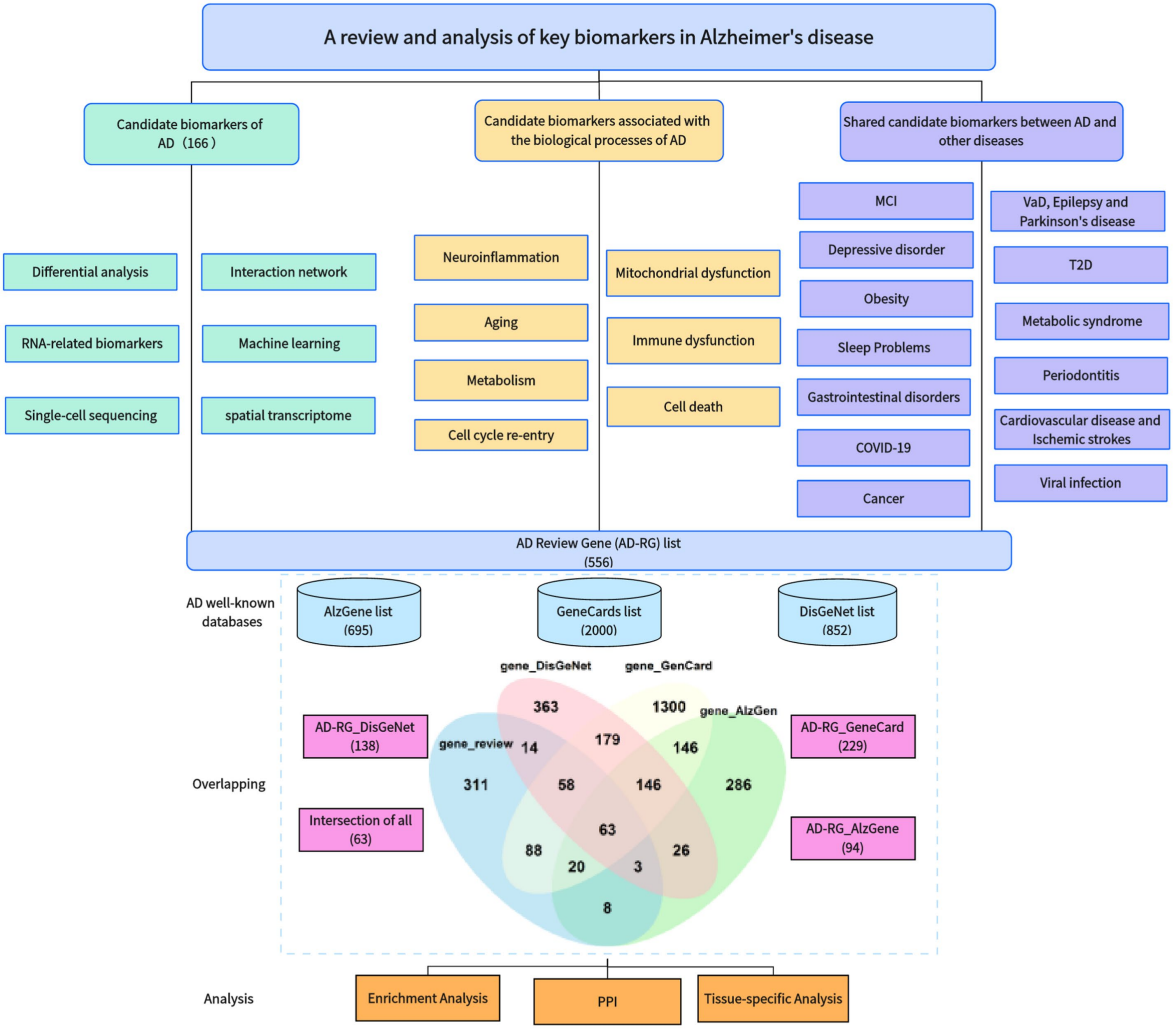
Article workflow diagram.

In recent years, we have noticed that a large number of studies have been devoted to identification of key AD biomarkers, including many previously unreported genes that have been identified as hub genes. The workflow diagram of this article is shown in Figure 1. In order to gain a more intuitive understanding of the achievements in this research field, this article covers the following work:

- We have reviewed approximately 180 papers on the identification of key AD genes, including AD biomarker genes, AD genes combined with biological processes, and pleiotropic genes underlining AD and related diseases.
- Based on previous research results, we constructed an AD Review Gene (AD-RG) list including 565 genes and compared it with well-known disease databases to obtain a list of shared genes.
- We also performed enrichment analysis and protein interaction analysis on these gene lists and studied the tissue specificity of these genes’ connectedness within the GTEx database.

## 2 Methods of identification of candidate biomarkers for AD

A significant amount of research effort has been devoted to the identification of candidate biomarkers for AD and its diagnosis, such as fine genetic mapping using genome-wide association studies (GWAS), traditional statistical studies, and recent ones employing network analysis of gene interactions, and machine learning algorithms.

GWAS research provides insights into various biological processes involved in AD. However, the challenge lies in interpreting the functional implications of genetic variants. To address this, an increasing number of studies have emerged that combine genetic data with gene expression data to elucidate the mechanisms underlying AD (Bihlmeyer et al., 2019). Table 1 lists the genes reported by GWAS-related studies. Jonas et al. (2022) identified variants in many genes related to immunity and/or microglia, using whole-genome sequencing and GWAS analysis. Liu A. et al. (2022) applied an Edge-Weighted Dense Module Search of GWAS, to integrate AD GWAS statistics of 472,868 individuals with proteomic profiles from parahippocampal gyrus (PHG), and dorsolateral prefrontal cortex (DLPFC), and pinpointed three potential drug target genes. Baird et al. (2021) used Mendelian randomization and colocalization, two methods that combination exploit these genetic variants to estimate the causal effects of individual genes, and identified 5 potential AD therapeutic targets. Novikova et al. (2021) integrated AD GWAS data with myeloid-specific epigenomic and transcriptomic datasets, and identified 11 genes as risk factors for AD to 20 loci. Sun Y. F. et al. (2022) utilized two gene expression prediction models of blood to predict meta-GWAS data, determining the expression of 108 genes in blood associated with AD risk, and identified 15 differentially expressed genes (DEGs). Kosoy et al. (2022) employed transcriptional and chromatin accessibility analysis on primary human astrocytes derived from 150 donors to identify putative regulatory mechanisms of 21 AD risk loci. Within these loci, 18 were further refined to single genes, including three novel candidate risk genes.

**Table 1:** AD genes from GWAS-related studies.

Reference	Data source	Genes
Raj et al. (2018)	Large-scale GWAS summary data provided by IGAP with total 17,008 AD cases and 37,154 controls, include 7,055,881 SNPs, we selected 6,004,159 SNPs	MLH3, FNBP4, CEACAM19, MLH3
Baselmans et al. (2019)	Deep sequencing data in the DLFPC of 450 subjects from two aging cohorts.	AP2A1, AP2A2, FUS, MAP1B, TBC1D7, ABCA7, RHBDF1, VPS53
Bihlmeyer et al. (2019)	Review related to immunity and microglia	TREM2, CD33, APOE, APII, MS4A, ABCA7, BIN1, CLU, CR1, INPP5D, PICALM, PLCG2
Baird et al. (2021)	Accelerating Medicines Partnership for Alzheimer’s Disease consortium (AMP-AD) and the Common Mind Consortium (CMC) meta-analysis study data (*n* = 1,286)	ACE, GPNMB, KCNQ5, RERE, SUOX
Novikova et al. (2021)	Epigenomic annotations using the International Genomics of Alzheimer’s Project (IGAP) AD GWAS datase. Schizophrenia SNP heritability (using the Psychiatric Genomics Consortium SCZ GWAS dataset as control)	AP4E1, AP4M1, APBB3, BIN1, MS4A4A, MS4A6A, PILRA, RABEP1, SPI1, TP53INP1, ZYX
Liu A. et al. (2022)	472,868 individuals with proteomic profiles from PHG, and DLPFC	APP, SNCA, VCAM1
Sun Y. F. et al. (2022)	AD GWAS data involving 71,880 (proxy) cases and 383,378 (proxy) controls of European ancestry from three consortia (Alzheimer’s disease working group of the Psychiatric Genomics Consortium (PGCALZ), IGAP and the Alzheimer’s disease sequencing project (ADSP)) and the UK Biobank data	HP1BP3, CD2AP, TMEM170B, NRF1, CCDC6, PICALM, CYP11A1, KAT8, RNF40, VKORC1, YPEL3, ACE, EPG5, BLOC1S3, KLC3
Kosoy et al. (2022)	The ATAC-seq (*n* = 107), RNA-seq (*n* = 127), SNP array (*n* = 122), and Hi-C (*n* = 5) data were generated from human brains of 150 individuals from four biobank resources (three based in New York City, NY, and ROSMAP from Rush University, Chicago, IL), including 123 autopsies and 27 biopsies.	KCNN4, FIBP, LRRC25
Jonas et al. (2022)	Review of whole genome sequencing and GWAS analyses identified variants in immune-and/or microglia-related genes	TREM2, CD33, APOE, API1, MS4A, ABCA7, BIN1, CLU, CR1, INPP5D, PICALM and PLCG2

These studies investigated the transcriptome-level mechanisms of AD in different tissues. The correlation between GWAS loci and transcriptome regulation is typically explored through expression quality loci (eQTLs) analysis. eQTLs serve as connections between GWAS loci and disease susceptibility (Albert and Kruglyak, 2015). The integration of extensive gene expression data from specific tissues with GWAS data related to the disease has led to the development of transcriptome-wide association studies (TWAS). This innovative approach has been recognized as a potent method for identifying genes that exhibit significant associations between their expression in specific tissues and the disease of interest (Gamazon et al., 2015; Gusev et al., 2016; Barbeira et al., 2018). Hao et al. (2019) collected a big sample of 17,008 AD cases and 37,154 controls to construct gene expression prediction models in various tissues including the DLPFC, adipose tissue, and blood tissue, and identified 4 new genes. Raj et al. (2018) utilized gene expression prediction models specifically for DLPFC tissue and identified eight associated genes at novel loci through the analysis of 25,580 cases and 48,466 controls. Leveraging a newly developed TWAS framework called UTMOST, Baselmans et al. (2019) conducted a comprehensive analysis and revealed 126 tissue-specific associations involving 50 unique genes.

### 2.1 Differential analysis

One of the simplest approaches for identifying potential biomarkers is to search for differential data between different phenotypes. These differential data can help researchers understand the mechanisms underlying diseases, develop diagnostic and treatment approaches, and address issues related to individual variations and biological diversity. For example, Madar et al. (2021) constructed six different classifiers to distinguish between healthy and diseased samples using 26 differentially expressed genes (DEGs). The DEGs were identified through their statistical *p*-values from the differential expression (DE) tests and further analyzed using the online annotation tool DAVID. The researchers used these DEGs as features in their classification models to distinguish diseased samples from healthy ones.

### 2.2 Interaction network

Traditional DE analysis typically emphasizes genes with significant expression level changes under different conditions but overlooks the complex interactions and regulatory mechanisms between genes. People are gradually realizing that complex biological phenomena cannot be solely analyzed at a single level, but require the integration of different components within the system for comprehensive study. Genes interacting with each other may cause perturbation in the molecular pathways leading to complex diseases. Genes, RNA, proteins, metabolites, and their internal and external interactions collectively form a complex system, and these interactions facilitate the functioning of cells. More and more databases are also incorporating corresponding molecular networks, including protein–protein interaction (PPI) networks, metabolic networks, and gene regulatory networks. The establishment and analysis of these networks improve our understanding of the complexity of biological systems and the interrelationships among biomolecules.

PPI plots are an effective method to bridge the gap between mRNA-based gene expression findings and protein-level interactions. Gui et al. (2021) obtained DEGs by differential analysis and identified GAPDH, RHOA, RPS29, and RPS27A as candidate genes for AD by constructing a PPI network. Yang et al. (2020) analyzed the transcriptome microarray of the prefrontal cortex (PFC) between AD specimens and non-AD controls and screened ten hub genes. Li et al. (2021) identified 10 hub genes in the entorhinal cortex (EC) and hippocampus (HIP) of patients with AD. Pang et al. (2017) found some functional hub genes from microarray data of EC and HIP of AD. Xu et al. (2022) identified 10 hub genes were the most targeted DEGs in the miRNA-mRNA network, and TIMP1, HLA-DRA, VWF, and FGF2 were the top four targeted DEGs in the TF-gene network. Rahman et al. (2019b) constructed TF-DEGs and miRNA-DEGs interaction networks (Table 2).

**Table 2:** The hub genes list by PPI method.

Reference	Data source	Genes
Pang et al. (2017)	GSE5281, GSE48350	ErbB2, ErbB4, OCT3, MIF, CDK13, GPI
Rahman et al. (2019b)	GSE4757	UBA52, RAC1, CREBBP, AR, RPS11, SMAD3, RPS6, RPL12, RPL15, UBC
Yang et al. (2020)	GSE36980	PDHA1, CLTC, YWHAE, MAPK6, YWHAZ, GRB2
Li et al. (2021)	GSE5281	GPI, PYGB, PFKM, ATP5C1, ATP5B, ATP6V1E1, LDOC, ATP6VOD1, ENO1, ATP6V1H
Gui et al. (2021)	GSE63061	GAPDH, RHOA, RPS29, RPS27A
Xu et al. (2022)	GSE11882	C1QC, C1QA, C1QB, CD163, FCER1G, VSIG4, CD93, CD14, VWF, CD44

Weighted gene co-expression network analysis (WGCNA) is a powerful screening tool that constructs a scale-free gene co-expression network to explore the relationship between genes with similar expression patterns and external clinical information (Langfelder and Horvath, 2008). Zhang T. et al. (2021) utilized DE analysis and WGCNA analysis to identify 16 hub genes associated with AD. Xia et al. (2022) obtained DEG-enriched co-expression networks between AD and normal samples in multiple transcriptomics datasets by WGCNA and found GJA1 interacts with AD from target-drugs-diseases network prediction. Li and De Muynck (2021) identified AD hub genes by PPI and WGCNA, which are enriched in microglial genes. Zhang F. et al. (2020) identified AD hub genes by WGCNA, including WDR47, OXCT1, C3orf14, ATP6V1A, SLC25A14, and NAPB. Among them, three hub genes (ATP6V1A, SLC25A14, OXCTI) may contribute to AD pathogenesis through the pathway of the TCA cycle (Table 3).

**Table 3:** AD hub genes list by WGCNA method.

Reference	Data source	Genes
Liang et al. (2018)	GSE1297	MT1, MT2, MSX1, NOTCH2, ADD3, RAB31
Su et al. (2019)	GSE48350. GSE5281, GSE26927, GSE5281, GSE36980, GSE29378, GSE48350, GSE1297, GSE5281, GSE84422	GAPDH, RPS27A, GFAP, B2M, CLU, EEF2, GJA1, CP
Zhu M. et al. (2020)	GSE122063, GSE36980, GSE5281, GSE118553, GSE132903, GSE106241, GSE63060, GSE63061	AP3B2, GABRD, GPR158, KIAA0513, MAL2
Zhang F. et al. (2020)	GSE36980, GSE 1297 GSE28146	WDR47, OXCT1, C3orf14, ATP6V1A, SLC25A14, NAPB
Li and De Muynck (2021)	The Banner Sun Health Research Institute under its brain donation program (STG, *n* = 76), (IFG, *n* = 65)	TREM2, C3AR1, ITGAX, OLR1, CD74, HLA-DRA, CDK2AP1
Zhang T. et al. (2021)	GSE5281	ATP5C1, PSMD1, ATP5B, EIF3H, EMC4, PSMB7, RAD51C, FAM162A, RAP1GDS1, BRAF, NME1, AP3M2, RRAGA, BLVRA, PSMD4, ATP6V1H
Xia et al. (2022)	AlzData (http://www.alzdata.org/) and ADNI data (http://adni.loni.usc.edu)	GJA1
Santiago et al. (2023)	GSE1297; GSE118553; GSE109887	ENO2, ELAVL4, SNAP91, NEFM

### 2.3 Machine learning

Researchers are also dedicated to using artificial intelligence (AI), machine learning (ML), and deep learning (DL) algorithms for detecting AD and integrating different types of data (Alamro et al., 2023). These data types include, but are not limited to, neuroimaging data, non-coding RNAs, transcriptomic data (Qorri et al., 2020), miRNA biomarkers (Xu et al., 2022), or other genomic data (Monk et al., 2021). These advanced computational techniques enable the analysis and integration of diverse data sources, allowing for a more comprehensive understanding of AD and improving diagnostic accuracy and prediction models. Yu et al. (2021), employed the Least Absolute Shrinkage and Selection Operator (LASSO) feature selection method for DEGs related to AD. Abyadeh et al. (2022) analyzed gene expression datasets from different brain regions using the robust rank aggregation (RRA) method, and predicted three miRNAs, namely hsa-mir-17-5p, hsa-mir-106a-5p and hsa-mir-373-3p, as potential candidates targeting these genes. Three transcription factors (TFs) ELK-1, GATA1, and GATA2 were also identified as the potential upstream regulators of the robust DEGs. Zhu et al. (2023b) identified TAC1 as a hub gene using the RRA method, which may be associated with synaptic function and inflammation. Zhu et al. (2023a) utilized a robust rank aggregation method to determine 1,138 differently expressed genes associated with AD. They performed WGCNA, LASSO, and logistic regression to investigate 13 hub genes exhibiting a high enrichment in immune function. Yu et al. (2021) identified BDNF and WWTR1 as critical genes of AD by LASSO and logistic regression, which are associated with the Braak stage, A beta 42 levels, and beta-secretase activity. Guo et al. (2023) identified 10 hub genes and found neuroinflammation and T-cell antigen receptor (TCR)-associated genes (LCK, ZAP70, and CD44) were the top three hub genes and validated using machine learning. Liu C. et al. (2023) collected transcriptomic data from the hippocampus to investigate the impact of immune cell infiltration on AD. It was found that monocytes are important immune cells associated with AD, and four genes related to AD and monocytes were identified: KDELR1, SPTAN1, CDC16, and RBBP6. Additionally, the research results demonstrate the involvement of KDELR1, SPTAN1, CDC16, and RBBP6 in lipid metabolism and immune response. Sekaran et al. (2023) conducted prioritized gene clustering analysis using the STRING database and trained candidate gene biomarkers using various supervised ML classification algorithms. They identified ORAI2 as a closely associated biomarker with the progression of AD. Additionally, within the ORAI2 gene network, they found three hub genes, namely TPI1, STIM1, and TRPC3, which may potentially be involved in the molecular pathogenesis of AD. Jin et al. (2023) proposed an improved DL algorithm called Differential Gene Selection TabNet (DGS-TabNet) for binary and multi-class classification of AD. The algorithm demonstrated excellent performance compared to five classical ML methods. They identified AVIL and NDUFS4 genes as important global genetic features. Lai et al. (2022) utilized unsupervised clustering to estimate subgroups of the immune microenvironment. In AD patients, the immune microenvironment was found to consist of two subgroups, one of which was associated with the metabolic phenotype and belonged to the immune-active type. Duan et al. (2022) screened five AD hub genes by WGCNA, and logistic regression analysis further identified ATP2A2, ATP6V1D, CAP2, and SYNJ1 were hub genes. Liu Z. et al. (2021) identified seven genes by LASSO and SVM-RFE (Table 4).

**Table 4:** The hub genes list by machine learning method.

Reference	Data source	Method	Genes
Liu Z. et al. (2021)	GSE63061, GSE85426	LASSO, SVM-RFE	ABCA2, CREBRF, CD72, CETN2, KCNG1, NDUFA2, and RPL36AL
Yu et al. (2021)	GSE33000, GSE36980, GSE48350, GSE5281, GSE122063, GSE106241, GSE4226, GSE97760, GSE63060, GSE63061	LASSO regression	BDNF, WWTR1
Abyadeh et al. (2022)	GSE118553, GSE44768, GSE48350, GSE5281, GSE33000, GSE44770, GSE36980, GSE122063, GSE132903, GSE29378	RRA	ELK-1, GATA1, GATA2
Duan et al. (2022)	GSE1297 GSE28146 GSE36980	Logistic regression	ATP2A2, ATP6V1D, CAP2, SYNJ1
Zhu et al. (2023a)	GSE118553, GSE122063, GSE36980, GSE48350, GSE5281, GSE36980, GSE48350, GSE5281, GSE118553, GSE122063, GSE36980, GSE132903, GSE5281, GSE140829, ADNI dataset	RRA, LASSO regression	CD163, CDC42SE1, CECR6, CSF1R, CYP27A1, EIF4E3, H2AFJ, IFIT2, IL10RA, KIAA1324, PSTPIP1, SLA, TBC1D2, APOE
Zhu et al. (2023b)	GSE118553, GSE122063, GSE36980, GSE33000, GSE48350, GSE44770, GSE5281	RRA	TAC1
Guo et al. (2023)	GSE173955, GSE203206, GSE15222, GSE97760	random forest (RF) binary classifier, Gaussian mixture model (GMM), linear model (LM), and support vector machine (SVM)	LCK, ZAP70, CD44, CD2, SNAP25, CD3E, CXCL8, HIST1H3J, IL12RB2, STAT4
Liu C. et al. (2023)	GSE5281, GSE48350	logistic regression and RF	KDELR1, SPTAN1, CDC16, RBBP6
Sekaran et al. (2023)	GSE36980	Logistic Regression (LR), RF, Linear Support Vector Machines (L-SVM), Naive Bayes (NB), and Multilayered Perceptron Neural Network (MLP-NN)	ORAI2, TPI1, STIM1, TRPC3
Jin et al. (2023)	GSE63060	DGS-TabNet	AVIL, NDUFS4
Duan et al. (2022)	GSE1297 GSE28146 GSE36980	Logistic regression	ATP2A2, ATP6V1D, CAP2, SYNJ1

### 2.4 Single-cell sequencing and spatial transcriptome

With the rapid advancement of sequencing technology, more detailed single-cell sequencing data and spatial transcriptome data provide richer information for advertising marker screening. Traditional gene expression analysis is usually performed at the cell population level, while single-cell sequencing technology can reveal the heterogeneity between cells and the characteristics of individual cells, allowing researchers to identify and classify cell types, discover cell subpopulations, and study cells. State and dynamic changes, as well as revealing intercellular interactions and communication networks. Spatial transcriptome technology combines gene expression information with tissue structure to obtain spatial distribution information of gene expression while maintaining the integrity of tissue structure. This allows researchers to understand gene expression patterns in tissues, the distribution of different cell types, and interactions between cells.

Chen Y. et al. (2022) have discussed that monocytes are important mediators in the prevention of AD development through exercise. Single-cell transcriptome analysis found that CD14+ and CD16+ monocytes interact with other cells in the circulating blood. Key ligand receptor-related genes TNF, CCR1, APP, and AREG are differentially expressed between exercise-treated and AD patients. Recent research shows that the hippocampus plays an important role in conditioned fear memory (CFM). Shen et al. (2023) used single-cell RNA sequencing (scRNA-seq) technology and found that CA subtype 1 has characteristic gene markers Ttr and Ptgds, which are speculated to be the result of acute stress and promote the production of CFM. Sirkis et al. (2023) explores the association of early-onset Alzheimer’s disease (EOAD) with specific peripheral immune signatures, and single-cell RNA sequencing identified significant expansion of a CD4 T cell termed interferon (IFN) signaling-associated gene (ISAG) hi T cells. Lin et al. (2023) has explored the association between high-fat diet and AD and T2D. Analysis of single-cell RNA-seq (scRNA-seq) data indicated C4b is astrocyte-specific. Spatial transcriptomics (ST) revealed C4b colocalizes with Gfad, a known astrocyte marker, and the colocalization of C4b expressing cells with Gad2 expressing cells, i.e., GABAergic neurons, in mouse brain. Chen S. et al. (2022) By applying Visium to human MTG spatial transcriptome profiles of AD and control cases, not only layer-specific markers shown in other studies (RORB, PCP4, MBP) were identified, but also new marker genes that have not been reported (SPARC, CALB2, DIRAS2, KRT17).

### 2.5 RNA-related biomarkers

In addition to different identification methods, many studies have identified RNA-related biomarkers. The interaction between differential long noncoding RNA (lncRNA) and/or targeted microRNA (miRNA) and messenger RNA (mRNA) has been demonstrated in AD and AD pathogenesis. Zhang et al. (2022b) identified four AD genes and two miRNAs and constructed a network in which miRNAs and transcription factors jointly regulate pathogenic genes. Zhang T. et al. (2020) integrated genomic transcriptome datasets through biomolecular networks, found 9 genes, as well as 6 common transcription factors, 10 miRNAs, and identified 10 AD candidate biomolecules by protein-drug interactions. Zhao et al. (2016) mapped DEGs to the target genes to construct miRNA-regulated networks, target genes SEC22B, RAB10, and FLT1 may be potential biomarkers of AD. Quan et al. (2020) identified 8 genes and 2 hub miRNAs from hippocampus microarrays data by PPI and miRNA-target network. Huang Z.-H. et al. (2022) constructed an mRNA-miRNA network and discovered six hub genes. Wang et al. (2020) construct the AD specific miRNA-mRNA network and identified five key miRNAs by topological analysis.

In the research on AD, lncRNAs play a crucial role. Yan et al. (2019) identified hub genes from the hippocampus, including CDC42, BDNF, TH, PDYN, VEGFA, CALB, CD44, TAC1, and CACNA1A, as well as OXT and TAC1 from the entorhinal cortex, and identified linc00622, linc00282, and linc00960 as novel potential candidates participating in the pathological mechanism of AD. Based on the competing endogenous RNA (ceRNA) hypothesis, Zhang J.-J. et al. (2021) constructed a lncRNA-miRNA-mRNA network and identified lncRNAs MALAT1, OIP5-AS1, LINC00657, and lnc-NUMB-1 as regulators of key pathogenic genes in AD such as APP, PSEN1, and BACE1. These lncRNAs may facilitate the distribution of β-amyloid protein (A-protein) in the brain, potentially through exosomes. This type of systems biology algorithm partially overcomes the limitation of traditional research that focuses only on differential gene expression while neglecting the high correlation between genes. Zhang et al. (2022a) identified 10 key AD genes, led by MAPT and AP2M1 by the mediation center algorithm, and used hub circRNAs and mRNAs to develop ceRNA networks (Table 5).

**Table 5:** Genes and RNAs list.

Reference	Data source	Genes	RNAs
Zhao et al. (2016)	GSE16759	SEC22B, RAB10, FLT1	miRNA-206, miRNA-655, miRNA-30e-3p, miRNA-369-3p
Zhou et al. (2019)	Accelerating Medicines Partnership-Alzheimer’s Disease (AMP-AD)	-	hsa-miR-155-5p, CERS6-AS1, and CTB-89H12.4
Yan et al. (2019)	GSE48350	CDC42, BDNF, TH, PDYN, VEGFA, CALB, CD44, TAC1, and CACNA1A	linc00662, linc00282 linc00960
Quan et al. (2020)	GSE5281, GSE48350	YWHAZ, DLG4, AGAP2, EGFR, TGFBR3, PSD3, RDX, BRWD1	hsa-miR-106b-5p and hsa-miR-93-5p
Zhang T. et al. (2020)	GSE4226, GSE4229	NOL6, ATF3, TUBB, UQCRC1, CASP2, SND1, VCAM1, BTF3, VPS37B	mir-20a-5p, mir-93-5p, mir-16-5p, let-7b-5p, mir-7085p, mir-24-3p, mir-26b-5p, mir-17-5p, mir-193-3p, mir-186-5p
Wang et al. (2020)	GSE63060, GSE63061 GSE18309	-	hsa-miR-93, hsa-miR-26b, hsa-miR-34a, hsa-miR-98-5p and hsa-miR-15b-5p
Ma et al. (2021)	GSE16759, GSE28146	ADAMTS1, CITED2, and GABRA2.	miR-548c-3p
Zhang J.-J. et al. (2021)	GSE5281, GSE48350, GSE9770, and GSE28146.	APP, PSEN1, BACE1	lncRNAs MALAT1, OIP5-AS1, LINC00657, lnc-NUMB-1
Dobricic et al. (2022)	Oxford Brain Bank	GABRB1, HCFC2, SLC16A3.	MiR-129-5p, miR-132-5p, miR-138-5p
Zhang et al. (2022b)	GSE1297, GSE5281	TBP, CDK7, GRM5, and GRIA1	hsa-miR-425-5p, hsa-miR-186-5p
Huang Z.-H. et al. (2022)	GSE33000, GSE48552, GSE36980, GSE28146, GSE147232 GSE159699	CALN1, TRPM7, ATR, SOCS3, MOB3A and OGDH	-
Qu et al. (2022)	GSE28146	-	LINC02047, LINC01124, LINC02478. miR-4060, miR-4090, miR-4786, miR-3612, miR-1254, miR-132.
Cai Z. et al. (2022)	Transgenic mice with five familial AD mutations.	-	lncRNA ENSMUST00000127786
Zhang et al. (2022a)	GSE5281, GSE122603, GSE97760, GSE150693, GSE1297, GSE161435	MAPT, AP2M1, ATР6V0C, SNCA, ATP6V1G2, ATP2B3, TTN, LMO7, SYNE1, AHNAK	has_circ_002048

## 3 Identification of candidate biomarkers associated with the biological processes of AD

Application of bioinformatics methodologies in target identification can be categorized as ascertaining pathways involved and targets associated with the genetic, epigenetic, and transcriptomic factors of AD (Singh et al., 2022). The pathogenesis of AD involves numerous cellular processes, including immune inflammation, cholesterol metabolism, apoptosis, synaptic dysfunction, and oxidative stress. Elucidating the specific molecules, the exact underlying molecular mechanisms and the pathways help to comprehend the pathogenesis and identify the therapeutic targets of the disease (Liu N. et al., 2021).

### 3.1 Mitochondrial dysfunction

Mitochondrial dysfunction is closely linked to the core pathological feature of AD: neuronal dysfunction (Ashleigh et al., 2023). Castora et al. (2022) conducted a qPCR analysis of gene expression for 84 genes involved in mitochondrial biogenesis. They found 9 hub genes involved in various aspects of mitochondrial function and regulation, including protein transport to mitochondria, mitochondrial morphology, maintenance of mitochondrial membrane potential, mitochondrial fragmentation and dysfunction, amyloidosis, and neuronal cell death. Chen F. Q. et al. (2022) found 5 central genes related to mitochondrial complexes. Enrichment analysis of these hub genes revealed disruptions in mitochondrial complexes in the context of AD pathogenesis. Zhang et al. (2023a) used PPI network, random forest, and two machine learning algorithms to obtain hub mitochondrial-related differentially expressed genes (MitoDEGs) closely related to AD. Zhao et al. (2023) identified six mitophagy-related hub genes (MRHGs) that be used as valuable diagnostic biomarkers for AD.

### 3.2 Oxidative stress

Oxidative stress is an important contributor to the pathogenesis of AD (Zhou et al., 2023). The overproduction of reactive oxygen species observed in AD patients results in the loss of mitochondrial function, altered metal ion homeostasis, lipopolysaccharide metabolism disorder, reduced anti-oxidant defense, increased release of inflammatory factors, and the aggravation and accumulation of amyloid-beta and tau hyper-phosphorylation, which directly cause synaptic and neuronal loss and lead to cognitive dysfunction. Li et al. (2023) screened differentially expressed oxidative stress genes and identified 15 hub genes using WGCNA and PPI analysis. Validation in an external dataset confirmed the expression of 9 hub genes.

### 3.3 Aging

Aging, the strongest single risk factor for AD, has been implicated in the accumulation of somatic cell mutations in neurons. Soheili-Nezhad et al. (2021) simulated the theoretical possibility of gene-associated somatic cell mutations induced by aging, with the results suggesting that long gene-dependent synaptic damage may contribute to the pathogenesis of AD. Additionally, telomeres, which are DNA sequences that protect chromosomes from damage, have been found to shorten with age and are of interest in the context of AD. Telomere-related genes have been proposed to play a role in the pathogenesis of AD. Ruan et al. (2023) identified telomere-related genes associated with aging clusters in AD patients and explored their immunological characteristics. Furthermore, they established prediction models for AD and AD subtypes based on TRGs and validated them using artificial neural network analysis and nomogram models. Balmorez et al. (2023) presented statistically significant shared genetic characteristics of aging, longevity, and AD. The study discussed important genes involved in these pathways, including TP53, FOXO, SUMO, IL4, IL6, APOE, and CEPT. One component of lipid metabolism includes APOE isoforms, which are considered risk factors for LOAD and are also associated with lifespan (Sebastiani et al., 2019). Liu T. et al. (2023) identified four genes (MSD14, PEBP1, ITPKB, and ATF7IP) for AD diagnosis from differentially expressed senescence-related genes, and found that the drug Abemaciclib is a targeted drug for the treatment of age-related AD.

### 3.4 Immune dysfunction

In the past few decades, there has been increasing attention on the immune dysregulation in AD. Mishra and Brinton (2018) pointed out that the inflammatory immune response is a unifying factor linking various risk factors of AD. Furthermore, it has been found that multiple genes are involved in neuroinflammation and immune activation associated with the occurrence and progression of AD in detection models. Xu and Jia (2020) identified immune-related DEGs. Eighteen hub genes were identified through PPI network analysis. An AD immune-related ceRNA network was generated using tools such as StarBase, DiANA-LncBase, and the Human MicroRNA Disease Database (HMDD). Zhuang et al. (2023) used the CIBERSORT algorithm to identify differentially infiltrated immune cells (DIICs). Ten biomarkers associated with AD immune infiltration were identified by WGCNA and machine learning algorithms. Liu C. et al. (2022) utilized WGCNA to explore immune cells and key genes associated with AD. Through LASSO and RF screening, they identified 10 key genes. Song et al. (2022) explored the role of the peripheral immune system in the pathogenesis of AD. It discovered a significant increase and decrease in the proportion of neutrophils and B lymphocytes in the blood of AD patients. Differentially expressed genes in AD neutrophils were found to be enriched in several AD-related pathways, such as ATP metabolism and mitochondrial organization. Additionally, it was observed that AD risk genes, including CD33 and IL1B, exhibited significant enrichment in protein–protein interaction network modules related to leukocyte-cell activation, mitochondrial organization, and cytokine-mediated signaling pathways in neutrophils. Lai et al. (2022) made a significant discovery using machine learning algorithms, identifying five immune microenvironment-related genes that are closely associated with AD pathological biomarkers and demonstrating accurate prediction of AD progression. The outputs of the machine learning model were further explained using the SHAP and LiME algorithms, providing valuable insights into the interpretation of the model’s outputs.

### 3.5 Metabolism

Gu et al. (2022) obtained nine hub genes associated with iron metabolism and AD by DE analysis and WGCNA. Glutamine (Gln) metabolism plays a crucial role in tumors. Wu et al. (2023) reported the identification of four potential Gln-related genes associated with AD through WGCNA. The analysis of their biological functions emphasized their role in determining cell fate, atrioventricular canal development, and neuronal fate.

### 3.6 Cell death

Autophagy is the process of cell self-digestion (Liu L. et al., 2022). It swallows its own cytoplasmic contents and wraps them to form vesicles, then fuse with lysosomes to form autolysosomes, which play a degrading role. Autophagy was originally considered to be a large-scale and non-selective degradation system. But in recent years, it has been gradually revealed that autophagy can selectively degrade senescent organelles, error proteins, and other substrates, thereby maintaining the homeostasis of the cell environment. Xia et al. (2023) found cell necrosis-related genes BAX, IL18, and CYCS exhibited significant differences between AD patients and normal controls. Disulfidptosis, a newly discovered type of cell death, seems to be closely related to the occurrence of various diseases. Ma et al. (2023) identified 22 overlapping genes between AD and disulfidptosis-related genes, and 7 hub genes were further obtained through machine learning.

Zhang et al. (2022) analyzed differential expression patterns in the hippocampus of AD patients and discovered dysregulation of ferroptosis-related genes. PCBP2 and FTL were significantly upregulated in the AD hippocampus, while VDAC2, LPCAT3, GSS, ACSL4, and ACSL6 were significantly downregulated. The altered expression of iron death-related DEGs affected the infiltration of specific immune cell types. Non-coding RNAs (ncRNAs) are involved in ferroptosis and AD progression, Tan et al. (2023) identified 5 AD and ferroptosis-related hub genes, and constructed a novel ferroptosis-related signature models including mRNAs, miRNAs and lncRNAs. Deng et al. (2022) screen correlative ferroptosis-related genes (FRGs) in the progress of AD by logistic regression.

Abnormalities in copper metabolism can prevent the clearance of β-amyloid peptides and promote the progression of AD pathogenesis. Zhang et al. (2023) identified seven cuproptosis genes by WGCNA.

### 3.7 Cell cycle re-entry

Zhou et al. (2021) explored the cell cycle re-entry mechanism in AD and mature neurons. Through WGCNA analysis, ten genes exhibited the strongest association with AD. Cross-signaling pathways of signal receptors, such as glutamatergic synapse, long-term potentiation, PI3K-AKT, and MAPK, were involved (Table 6).

**Table 6:** AD hub gene list combined with biological process research.

Reference	Biological processes	Hub gene
Castora et al. (2022)	Mitochondrial dysfunction	TP53, SOD2, CDKN2A, MFN2, DNM1L, OPA1, FIS1, BNIP3, GAPDH
Chen F. Q. et al. (2022)		COX5A, NDUFAB1, SDHB, UQCRC2, UQCRFS1
Zhang et al. (2023a)		BDH1, TRAP1, OPA1, DLD, OPA1
Zhao et al. (2023)		CD44, SUCLA2, DLAT, ITGAX, PPARG, MYC
Li et al. (2023)	Oxidative stress	CCK, CNR1, GAD1, GAP43, NEFL, NPY, PENK, SST, TAC1
Balmorez et al. (2023)	Aging	TP53, FOXO, SUMOylation, IL4, IL6, APOE, CEPT
Liu T. et al. (2023)		MSD14, PEBP1, ITPKB, ATF7IP
Deng et al. (2022)		RAF1, NFKBIA, MOV10L1, IQGAP1, FOXO1
Xu and Jia (2020)	Immune dysfunction	B2M, FYN, PIK3R1, PIK3CA
Zhao et al. (2022)		CHGB, APLNR, FGF13, PAK1, SERPINA3
Lai et al. (2022)		CXCR4, PPP3R1, HSP90AB1, CXCL10, S100A12
Qian et al. (2022)		NFKBIA, CD4, RELA, CASP3, HSP90AA1
Liu C. et al. (2022)		ARMCX5, EDN3, GPR174, MRPL23, RAET1E, ROD1, TRAF1, WNT7B, OR4K2, ZNF543
Song et al. (2022)		CD33, IL1B
Zhuang et al. (2023)		CMTM2, DDIT4, LDHB, NDUFA1, NDUFB2, NDUFS5, RPL17, RPL21, RPL26, NDUFAF2
Gu et al. (2022)	Metabolism	ATP6V1D, ATP6V1G2, ATP6V1H, CYP26B1, FBXO34, PGRMC1, PLOD1, SNCA, TSPO
Wu et al. (2023)		ATP5H, NDUFAB1, PFN2, SPHKAP
Zhang et al. (2022)	Cell death	PCBP2, FTL, GSS, ACSL4
Deng et al. (2022)		RAF1, NFKBIA, MOV10L1, IQGAP1, FOXO1
Xia et al. (2023)		BAX, IL18, CYCS
Ma et al. (2023)		MYH9, IQGAP1, ACTN4, DSTN, ACTB, MYL6, GYS1
Tan et al. (2023)		EPT1, KLHL24, LRRFIP1, CXCL2 CD44
Zhang et al. (2023)		IFI30, PLA1A, ALOX5AP, A4GALT
Zhou et al. (2021)	Cell cycle re-entry	GRIN2A, GRIA2, CHRM1, GABRG2, PGRMC1, EPHA4, MAGED1, TNFRSF1B, TNFRSF1A, RXRA

## 4 Identification of shared candidate biomarkers between AD and other diseases

Numerous scholars have discovered close connections between AD and other illnesses, attempting to unravel the mechanisms underlying their interactions.

### 4.1 MCI

Neurodegenerative diseases affect over 1 billion people, accounting for approximately 15% of the global population, with at least 7 million deaths attributed to neurodegenerative diseases annually (Maiese, 2016). MCI is a cognitive state that lies between normal cognition and dementia. Longitudinal studies have shown that some MCI patients remain in the MCI state, while others progress to AD. The reasons behind these diverse transitions in MCI are still under investigation. Shigemizu et al. (2020) utilized blood microRNA expression profiles and genomic data from 197 Japanese patients with MCI to construct a prognostic prediction model based on the Cox proportional hazards model. They found PTEN as a gene with differential expression between MCI and AD among four significant hub genes (SHC1, FOXO1, GSK3B, and PTEN). Xue et al. (2020) obtained DEG profiles of MCI, AD, and late-stage AD patients from the GEO database. Enrichment analysis revealed functional associations of the genes with mitochondria and ribosomes. Wang X. et al. (2021) collected a comprehensive dataset consisting of 1,036 brain imaging features and 15,481 gene expression values from 180 MCI patients. They utilized the WGCNA approach to discover key features that influence the conversion of MCI to AD. These features included the thickness of the left paracentral lobule and sulcus (L.PTs), as well as the expression levels of CTCF, UQCR11, and WDR5B genes. Rantanen et al. (2022) analyzed transcriptomic data from olfactory neural sphere (ONS)-derived cells in MCI and AD patients. They discovered a significant difference in the expression of AKAP6, which is associated with cognitive function. Donaghy et al. (2022) examined the gene expression differences between AD and Dementia with Lewy Bodies (DLB) in both the MCI and dementia stages. They identified multiple DEGs, among which ANP32A was identified as a potential prognostic marker for AD. Enrichment analysis of the DEGs specific to the MCI-AD/AD comparison revealed an upregulation of immune and inflammatory responses. The cognitive decline caused by AD shares a significant overlap with the cognitive decline caused by cerebrovascular diseases, making it challenging to differentiate between them. Qin et al. (2023) utilized Mfuzz clustering and WGCNA to examine the shared DEGs and differentially expressed miRNAs between MCI and AD. The resulting miRNA-mRNA network highlighted the potential involvement of miR-6764-5p in the pathogenesis of MCI and AD through its targeting of RPL11 in the ribosomal pathway.

### 4.2 VaD, epilepsy, and Parkinson’s disease

Tian et al. (2022) conducted a screening of DEGs between AD and vascular dementia (VaD) and employed WGCNA to construct a VaD-AD-specific PPI network for analysis. Luo et al. (2022) identified REPS1 as a shared hub gene between VaD and AD, REPS1 was associated with the activation of pyruvate metabolism and inhibition of the Ras signaling pathway.

Epilepsy often manifests in AD and hastens its progression. Wu et al. (2022) utilized WGCNA to perform co-expression analysis on the top 50% variably expressed genes in both AD and epilepsy datasets. They identified 229 and 1,187 genes in the key modules and determined that the co-regulatory factors for 17 overlapping genes were TF-Foxc1 and miRNA-hsa-mir-335-5p. Notably, the hub gene CXCR4 emerged as a potential target for 20 different drugs. Tang et al. (2023) identified 12 DEGs that were significant in AD and epilepsy, SCN2A, GRIA1, and KCNJ9 were the hub genes with high connectivity. Wang X.-D. et al. (2021) captured hub genes of AD- and epilepsy-associated gene co-expression modules by weighted key driver analysis.

Li et al. (2020) identified ATP1A1, ATP6V1G2, GOT1, HPRT1, MAP2K1, PCMT1, and PLK2 as key metabolic genes that were downregulated in AD, Parkinson’s disease (PD), and Huntington’s disease (HD), and screened 57 drugs that target these genes. Kelly et al. (2020) identified 12 shared SNPs between PD and AD. Gupta and Kumar (2021) identified 10 hub genes that be involved in the shared mechanism of AD and PD pathogenesis.

### 4.3 Depressive disorder

Cheng et al. (2021) investigated the underlying mechanisms linking AD and major depressive disorder (MDD) and identified 19 DEGs associated with both AD and MDD. Enrichment analysis revealed significant involvement of pathways related to circadian rhythm disruption and chronic depressive signaling. Through PPI and transcription factor (TF) and microRNA target gene network analysis, they identified five hub genes, namely DYNC1H1, MAPRE3, TTBK2, ITGB1, and WASL, which may serve as potential targets for diagnosis and treatment of both AD and MDD. Based on publicly available mRNA expression profile data, Song et al. (2023) identified differentially expressed immune-related genes (DEIRGs) involved in depression and AD. A total of 121 genes were found to be enriched in immune-related pathways, such as the JAK–STAT signaling pathway, chemotaxis regulation, chemotactic activity, cytokine-cytokine receptor interaction, and primary immunodeficiency. Through PPI network analysis, three hub genes, IL1R1, CHGB, and NRG1, were identified.

### 4.4 T2D

Cellular metabolic disorders, such as diabetes, have a widespread impact on various systems in the body, including the central nervous system, peripheral nervous system, inflammatory system, and vascular system (Maiese, 2023). Diabetes can lead to insulin resistance and dementia in patients with AD. It can affect stem cell proliferation, cell protective pathways, retinal diseases, and immune-mediated pathways involving microglial cells. Furthermore, over 70% of diabetes patients may develop peripheral neuropathy. Diabetes can cause autonomic neuropathy and peripheral nerve disorders. Recently, AD has been increasingly recognized as a brain-specific type of diabetes, referred to as type 3 diabetes. Several studies have indicated that individuals with type 2 diabetes (T2D) have a higher risk of developing AD (Chung et al., 2021). Chung et al. (2021) employed the non-negative matrix factorization method to generate gene clusters for AD and T2D, extracting common differentially expressed genes as candidate genes. These genes are enriched in pathways related to AD and T2D, such as T cell selection and chemokine signaling pathways. Yuan et al. (2023) screened a total of 175 shared genes between AD and T2D. These genes were found to be enriched in metabolic processes, lipid and atherosclerosis, AMPK signaling pathways, insulin resistance, chemokines, and cytokines. Zhu Y. et al. (2020) utilized WGCNA to mine GEO microarray data. The shared genes were found to be enriched in signaling pathways such as circadian rhythm, autophagy, glutathione metabolism, and synaptic vesicle cycle. Castillo-Velazquez et al. (2023) conducted a study to explore the shared gene and protein information between AD and DM2. A total of 1,551 common genes were obtained, and the hub genes were found to be enriched in biological processes and cytokine signaling pathways. Using the Metascape platform, 10 potential targets were identified, out of which 7 showed pharmacological interactions with monoclonal antibodies, anticancer drugs, and flavonoid derivatives currently in use. Gao et al. (2023) identified significant differentially expressed genes common to T2DM and AD by WGCNA. Molecular docking prediction showed that CD44 and STAT3 may play a significant role in the development of T2DM-induced AD. Lee and Lee (2021) constructed a co-expression network to identify COPS4, PSMA6, GTF2B, GTF2F2, and SSB as dysregulated transcription common factors between AD and DM. Ye et al. (2023) identified 10 shared hub genes between AD and T2D. Afzal et al. (2023) uncovered the mutual genomics motifs between AD and T2D via non-negative matrix factorization, and screened of six shared genes. Kang et al. (2022) constructed a PPI network consisting of AD and T2DM DEGs and found that the hub gene SLC2A2 (coding transmembrane carrier protein GLUT2), which connects the most DEGs in both AD and T2DM, plays a key regulator in linking T2DM and AD via glucose metabolism-related pathways. Shu et al. (2022) identified Five hub proteins between AD and T2D. Zhang et al. (2023b) identified seven hub genes of co-DEGs between T2DM, MDD, and dementia. Huang C. et al. (2022) confirmed through multiple comparisons that CACNA2D3, NUMB, and IER3 simultaneously participate in AD and T2D, and analyzed interacting chemicals, transcription factors, and miRNAs.

### 4.5 Obesity

Li T. et al. (2022) used WGCNA to define co-expression gene modules related to Obesity and AD. The functional analysis of shared genes emphasized that inflammation and mitochondrial function are common features of Obesity and AD pathophysiology. PPI analysis identified 6 hub genes, including MMP9, PECAM1, C3AR1, IL1R1, PPARGC1alpha, and COQ3, which were validated using qPCR.

### 4.6 Metabolic syndrome

Li J. et al. (2022) utilized WGCNA to identify co-expression gene modules shared between AD and metabolic syndrome. Candidate genes were identified using RF and LASSO, resulting in the identification of 8 diagnostic genes. Immunoinfiltration analysis was performed, and the ssGSEA results indicated significant regulation of immune-related genes in the glycolysis-metabolism pathway.

### 4.7 Sleep problems

Liang et al. (2022) conducted a multi-scale embedded gene co-expression network analysis to identify common DEGs between AD and sleep disorders and identified 10 hub genes. Wu et al. (2021) identified the hub gene between Obstructive sleep apnea syndrome and AD by WGCNA.

### 4.8 Periodontitis

Jiang et al. (2021) extracted and integrated shared DEGs between AD and periodontitis. These shared genes are associated with cell morphogenesis related to neuronal differentiation, leading-edge membrane, and receptor-ligand activity. PPI analysis identified 10 hub genes associated with AD. Jin et al. (2021) integrated AD-related genes with differentially expressed genes from periodontitis data. The shared genes were subjected to feature extraction using the Boruta algorithm and used to construct an SVM model. TF network and differentially expressed pathway network were constructed to determine the core common genes. Three TFs (FOS, MEF2C, and USF2) and several pathways (JAK–STAT, MAPK, NF-κB, and natural killer cell-mediated cytotoxicity) were identified as regulatory factors for these interacting genes. C4A, C4B, CXCL12, FCGR3A, IL1B, and MMP3 were identified as core shared hub genes.

### 4.9 Gastrointestinal disorders

Dong et al. (2023) identified PPARG and NOS2 are shared genes of AD and ulcerative colitis, They drive macrophages and microglia heterogeneous polarization, which may be potential targets for treating neural dysfunction induced by systemic inflammation. Adewuyi et al. (2022) conducted a comprehensive analysis of the relationship between AD and gastrointestinal disorders. The results showed significant genetic overlap and correlation between AD and gastroesophageal reflux disease, peptic ulcer disease, gastritis-duodenitis, irritable bowel syndrome, and diverticular disease, but not with inflammatory bowel disease. Seven shared genes were identified. Pathway analysis revealed significant enrichment of lipid metabolism, autoimmune response, lipase inhibitors, PD signaling pathway, and statin drug mechanisms, which are associated with the characteristics of AD and GiT.

### 4.10 Cardiovascular disease and ischemic strokes

Lee et al. (2021) constructed gene regulatory networks that utilized each of the AD and cardiovascular disease candidate disease-related gene sets and identified two common upstream genes (GPBP1 and SETDB2).

Rahman et al. (2019a) identified hub proteins that are shared between ischemic strokes and AD. Furthermore, protein-drug interaction analysis revealed that PDE9A interacts with drugs such as caffeine, γ-glutamyl glycine, and 3-isobutyl-1-methyl-7H-xanthine. Through the PPI and network topology analysis for the common DEGs, Liu W. et al. (2022) identified hub genes RPS3, RPS15, PSMB6, MRPL17, and MRPL24 of AD and IS.

### 4.11 COVID-19

Neuroinflammation and immune dysregulation play a crucial role in AD and are also associated with severe COVID-19 and neurological symptoms (Shi et al., 2023). The COVID-19 pandemic has caused millions of deaths and remains a significant global public health burden. Previous studies have found that a large number of COVID-19 patients and survivors experience neurological symptoms, making them a high-risk population for neurodegenerative diseases such as AD and PD. Genome-wide association studies have identified numerous risk single nucleotide polymorphisms for both AD and COVID-19. Khullar and Wang (2023) conducted an integrative multi-omics analysis, predicting gene regulatory networks in the major brain regions using AD population data. Machine learning analysis prioritized 36 AD-COVID candidate genes for predicting the severity of COVID-19. Shi et al. (2023) identify 52 common DEGs in COVID-19, AD, and PD, and found that these three diseases are involved in synaptic vesicle cycling and synaptic downregulation, suggesting that synaptic dysfunction may contribute to the occurrence and progression of neurodegenerative diseases caused by COVID-19. Wang et al. (2022) identify 40 DEGs that are shared between AD and COVID-19. These genes are enriched in the calcium signaling pathway and the PPI network.

### 4.12 Viral infection

Sun X. et al. (2022) investigated the relationship between viral infection and AD through bioinformatics analysis. By using WGCNA to detect DEGs, they identified 126 highly co-expressed modules. They further identified four central genes, TLR2, COL1A2, NOTCH3, and ZNF132, that are associated with both viral infection and AD. Talwar et al. (2019) identified 8 overlapping candidate genes from the retrieved APP, MAP, oxidative stress, inflammation, and aging-related high-confidence AD-related genes/proteins. The analysis revealed that APOE was mainly associated with hepatitis C virus, EGFR with the Epstein–Barr virus and Human papillomavirus, and APP and CASP8 with the Human herpes virus.

### 4.13 Cancer

Yılmaz (2020) found five genes as common DEG for five datasets of AD and cancer, EGFR for AD-breast cancer, SOX9 for AD-colorectal cancer, THBS1 for AD-lung cancer, and ‘VEGFA’ for AD-prostate cancer were identified as the most significant hub genes in network analysis. Cai J. et al. (2022) screened 13 hub genes of AD and Glioblastoma multiforme by seven typical algorithms co-expression networks. miRNAs are involved in the regulation of various cellular processes including pathological conditions. Petrovic et al. (2023) identified miR-107, miR-146a, and miR-17 as potentially good candidates for both AD and breast cancer treatment (targeting BRCA1/2 and PTEN in both diseases). Chen et al. (2021) constructed circRNA-miRNA target network for explore the circRNA relationship between AD and cancer, and found three hub nodes CircPICALM, circRTN4 and circMAN2A1. Zhang and Kiryu (2023) utilized WGCNA to identify 5 hub genes that were differentially expressed and associated with osteosarcoma. A diagnostic model was then established using LASSO. Drug-gene interaction databases were used to predict target drugs, and 78 drugs were predicted to target FOXO1, SP1, MAPK9, and BCL2, including fluorouracil, cyclophosphamide, and bortezomib (Table 7).

**Table 7:** AD gene list combined with relevant disease research.

Reference	AD related illnesses	Shared genes
Shigemizu et al. (2020)	MCI	SHC1, FOXO1, GSK3B, and PTEN
Xue et al. (2020)		RPS17, RPL26, RPS27A, RPS24, RPL31, EEF1B2, RPS27, TOMM7 RPL23
Wang X. et al. (2021)		CTCF, UQCR11 WDR5B
Rantanen et al. (2022)		AKAP6
Donaghy et al. (2022)		ANP32A
Qin et al. (2023)		RPL11
Tian et al. (2022)	VaD	SH3GL2, PROK2, TAC3, HTR2A, MET, TF, PTH2R, CNR1, CHRM4, PTPN3, CRH
Luo et al. (2022)		REPS1
Wu et al. (2022)	Epilepsy	CXCR4
Tang et al. (2023)		SCN2A, GRIA1, KCNJ9
Wang X.-D. et al. (2021)		TRPC1, C2ORF40, NR3C1, KIAA0368, MMT00043109, STEAP1, MSX1, KL, CLIC6
Li et al. (2020)	Parkinson’s disease	ATP1A1, ATP6V1G2, GOT1, HPRT1, MAP2K1, PCMT1 PLK2
Kelly et al. (2020)		EPB41L5, CYP26B1, IQCB1, DCPIA, CLGN, TDRD6, PSORSIC1, PARP12, WISP1, PIK3C2A, CLMN, DHX33
Gupta and Kumar (2021)		CDC42, CD44, FGFR1, MYO5A, NUMA1, TUBB4B, ARHGEF9, USP5, INPP5D, NUP93
Cheng et al. (2021)	Depressive disorder	DYNC1H1, MAPRE3, TTBK2, ITGB1, WASL
Song et al. (2023)		IL1R1, CHGB, NRG1
Yuan et al. (2023)	Diabetes	IL6, TNF, INS, IL1B, AKT1, VEGFA, IL10, TP53, PTGS2, TLR4
Zhu Y. et al. (2020)		CALM1, LRRK2, RBX1, SLC6A1, TXN, SNRPF, GJA1, VWF, LPL, AGT
Castillo-Velazquez et al. (2023)		STAT3, EGFR, IRS1, MAPK1, SRC, HSP90AA1, PIK3R1, UBC, MAPK3, ESR1
Gao et al. (2023)		CD44, STAT3
Lee and Lee (2021)		COPS4, PSMA6, GTF2B, GTF2F2, SSB
Ye et al. (2023)		NF1, RAB14, ADCY5, RAPGEF3
Afzal et al. (2023)		CDKN1A, COL22A1, EIF4A, GFAP, SLC1A1, VIM
Kang et al. (2022)		SLC2A2
Shu et al. (2022)		SFN, CD44, ITGB2, MERTK, GEM
Zhang et al. (2023b)		SMC4, CDC27, HNF1A, RHOD, CUX1, PDLIM5, TTR
Huang C. et al. (2022)		CACNA2D3, NUMB, IER3
Li T. et al. (2022)	Obesity	MMP9, PECAM1, C3AR1, IL1R1, PPARGC1alpha, COQ3
Li J. et al. (2022)	Metabolic syndrome	ARHGAP4, SNRPG, UQCRB, PSMA3, DPM1, MED6, RPL36AL, RPS27A
Liang et al. (2022)	Sleep Problems	ATP5A1, ATP5B, COX5A, GAPDH, NDUFA9, NDUFS3, NDUFV2, SOD1, UQCRC1, UQCRC2
Wu et al. (2021)		AREG, SPP1, CXCL2, ITGAX, DUSP1, COL1A1, SCD, ACTA2, CCND2, ATF3
Jiang et al. (2021)	Periodontitis	SPP1, THY1, CD44, ITGB1, HSPB3, CREB1, SST, UCHL1, CCL5, BMP7
Jin et al. (2021)		C4A, C4B, CXCL12, FCGR3A, IL1B, MMP3
Dong et al. (2023)	Ulcerative colitis	PPARG, NOS2
Adewuyi et al. (2022)	Gastrointestinal disorders	PDE4B, BRINP3, ATG16L1, SEMA3F, HLA-DRA, SCARA3, MTSS2, PHB, TOMM40
Lee et al. (2021)	Cardiovascular disease	GPBP1, SETDB2
Rahman et al. (2019a)	Ischemic strokes	PDE9A, GNAO1, DUSP16, NTRK2, PGAM2, MAG, TXLNA.
Liu W. et al. (2022)		RPS3, RPS15, PSMB6, MRPL17, MRPL24
Shi et al. (2023)	COVID-19	TAGLN3, GAD2, SST, SYP, KCNJ4.
Wang et al. (2022)		ITPR1, ITPR3, ITPKB, RAPGEF3, MFGE8
Sun X. et al. (2022)	Viral infection	TLR2, COL1A2, NOTCH3, ZNF132
Talwar et al. (2019)		AKT1, GSK3B, APP, APOE, EGFR, PIN1, CASP8, SNCA
Yılmaz (2020)	Cancer	CEBPD, DCN, DST, FHL1, SLIT3
Cai J. et al. (2022)		HPCA, CA10, PENK, CALB2, DRD2, CPNE6, SVOP, CCNA2, NCAPG, KIF20A, UBE2C, CKAP2L, NCAPH
Zhang and Kiryu (2023)		MAPK9, FOXO1, BCL2, ETS1, SP1

## 5 Discussion

### 5.1 Statistical analysis

The research work is divided into three modules, and 677 key genes are mentioned in this article. After deduplication statistics, we constructed a list of 565 key genes, called the AD Review Gene (AD-RG) list. Statistics on the number of occurrences of key genes are shown in Table 8. Notably, there are 20 genes (‘CD44’: 9 ‘GAPDH’: 5 ‘RPS27A’: 5 ‘APOE’: 4 ‘SNCA’: 4 ‘GJA1’: 4 ‘EGFR’: 3 ‘SST’: 3 ‘OPA1’: 3 ‘TP53’: 3 ‘ATP6V1G2’: 3 ‘TAC1’: 3 ‘ITGAX’: 3 ‘B2M’: 3 ‘GFAP’: 3 ‘CLU’: 3 ‘ATP6V1H’: 3 ‘ATP5B’: 3 ‘APP’: 3 ‘IL1B’: 3) has been reported more than 3 times in the literature.

**Table 8:** Frequency of gene occurrence in AD biomarker research.

Frequency	1	2	3	4	5	9
Count	486	59	14	3	2	1
Genes	-	-	EGFR, SST, OPA1, TP53, ATP6V1G2, TAC1, ITGAX, B2M, GFAP, CLU, ATP6V1H, ATP5B, APP, IL1B	APOE, SNCA, GJA1	GAPDH, RPS27A	CD44

In order to more accurately screen out the key genes of AD, this article obtained AD-related gene lists from multiple well-known databases, including AlzGene (Bertram et al., 2007), GeneCards, and DisGeNet (Pinero et al., 2020), and performed an intersection operation with the gene lists reviewed in this article to obtain overlapping genes. AlzGene is a well-known AD susceptibility gene database, currently containing 695 genes. The database was last updated in 2011. GeneCards is a widely used genetic database that provides a comprehensive genetic resource on human genes. We used “Alzheimer’s Disease” as the keyword to obtain the Relevance score and the top 2000 genes. The scoring rules of GeneCards are usually based on a comprehensive consideration of multiple factors, such as literature citations, experimental evidence, database annotations, and expert evaluations. DisGeNet is a widely used disease gene association database that provides comprehensive information on the associations between human diseases and genes. Using Alzheimer’s Disease as the keyword, 3,397 related genes were retrieved, and 852 genes with Score_gda greater than the average were retained.

**Figure 2:**
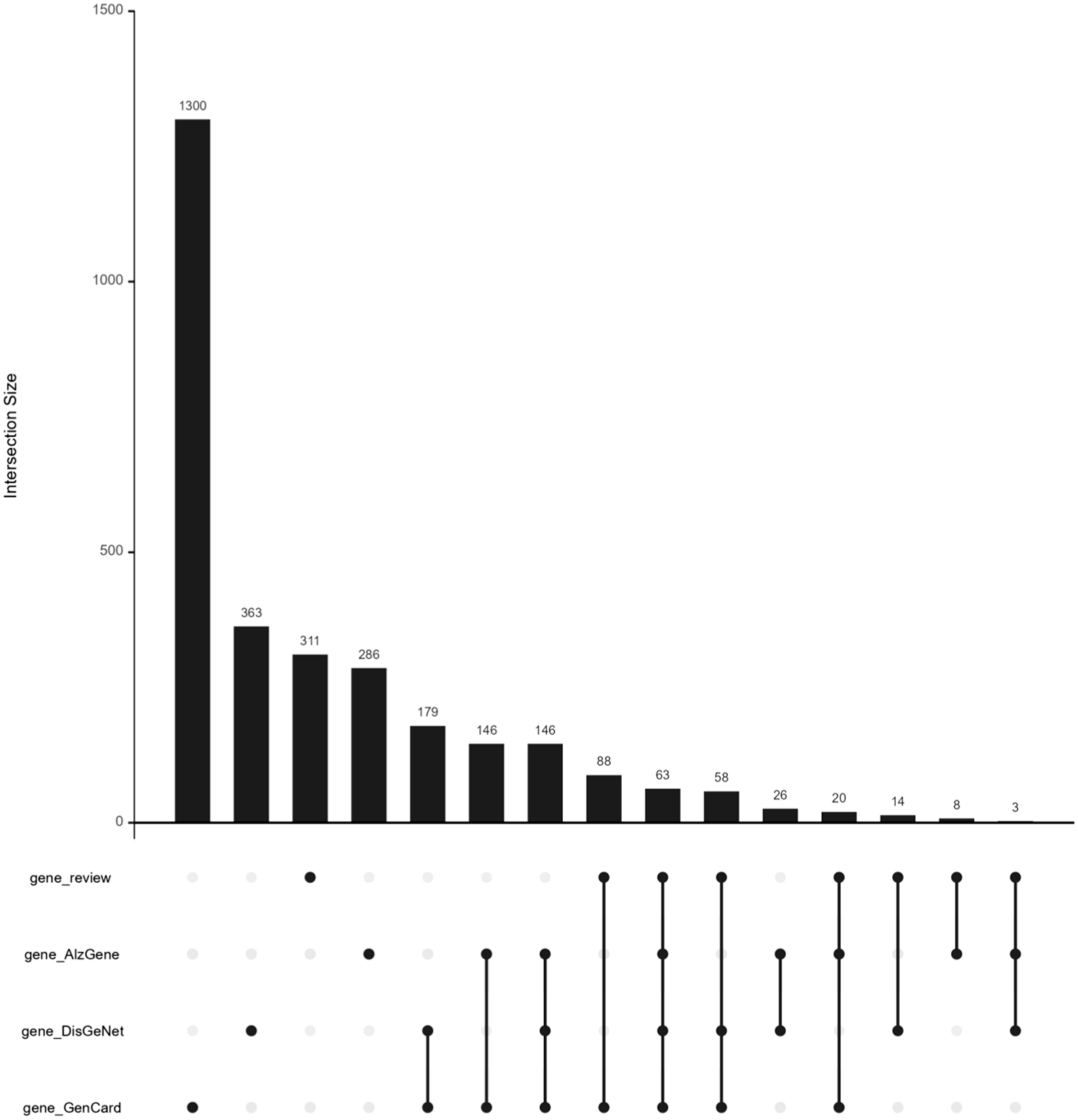
Upset plot of the four lists AD-RG, AlzGene, GeneCard, and DisGeNet.

The AD-RG list intersects with the three gene lists of AlzGene, GeneCard, and DisGeNet, respectively. AD-RG_AlzGene has 94 overlapping genes, AD-RG_GeneCard has 229 overlapping genes, AD-RG_DisGeNet has 138 overlapping genes, and the four gene lists have 63 overlapping genes. The upset plot formed by the intersection is shown in Figure 2.

### 5.2 Enrichment analysis

**Figure 3:**
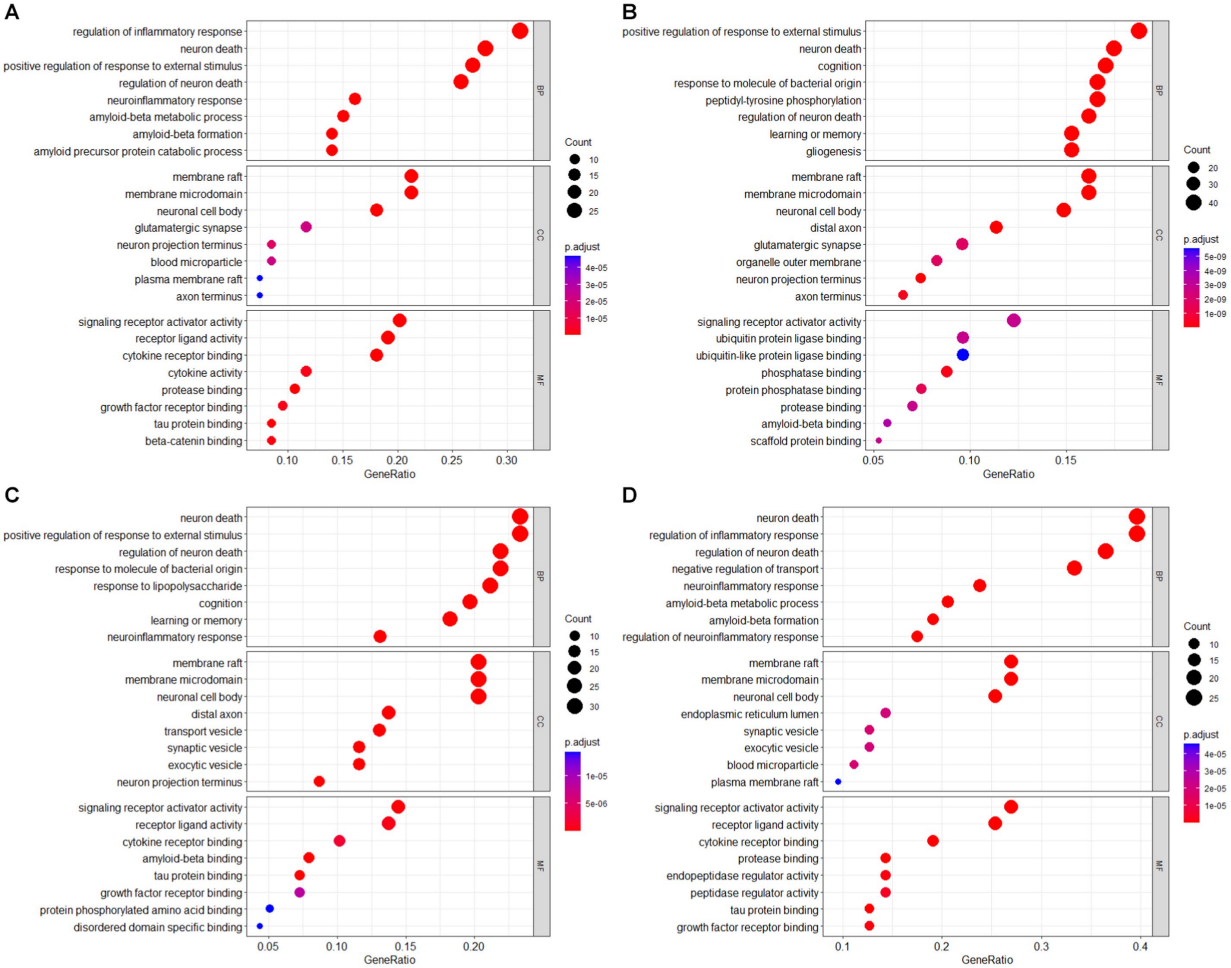
GO enrichment analysis bubble plot of AD gene list **(A)** AD-RG_AlzGene, **(B)** AD-RG_GeneCard, **(C)** AD-RG_DisGeNet, **(D)** intersection of all.

Enrichment analysis was conducted on the intersection results of the AD-RG_AlzGen, AD-RG_GeneCard, AD-RG_DisGeNet, and Intersection of all gene lists, which formed the AD gene list, in the field of bioinformatics. The bubble plot of the GO enrichment analysis is shown in Figure 3. Gene Ontology (GO) analysis revealed that the AD-RG_AlzGen gene list was significantly enriched in biological processes related to the regulation of inflammatory response, neuron death, regulation of neuron death, neuroinflammatory response, and amyloid-beta metabolic process. Similarly, the AD-RG_GeneCard gene list exhibited enrichment in biological processes associated with cognition, neuron death, learning or memory, positive regulation of response to external stimulus, and regulation of neuron death. Furthermore, the AD-RG_DisGeNet gene list showed significant enrichment in biological processes such as neuron death, positive regulation of response to external stimulus, requlation of neuron death, response to molecule of bacterial origin, and response to lipopolysaccharide. Finally, the intersection of all gene lists was enriched in neuron death, regulation of inflammatory response, regulation of neuron death, negative regulation of transport, and positive regulation of response to external stimulus.

### 5.3 PPI

**Figure 4:**
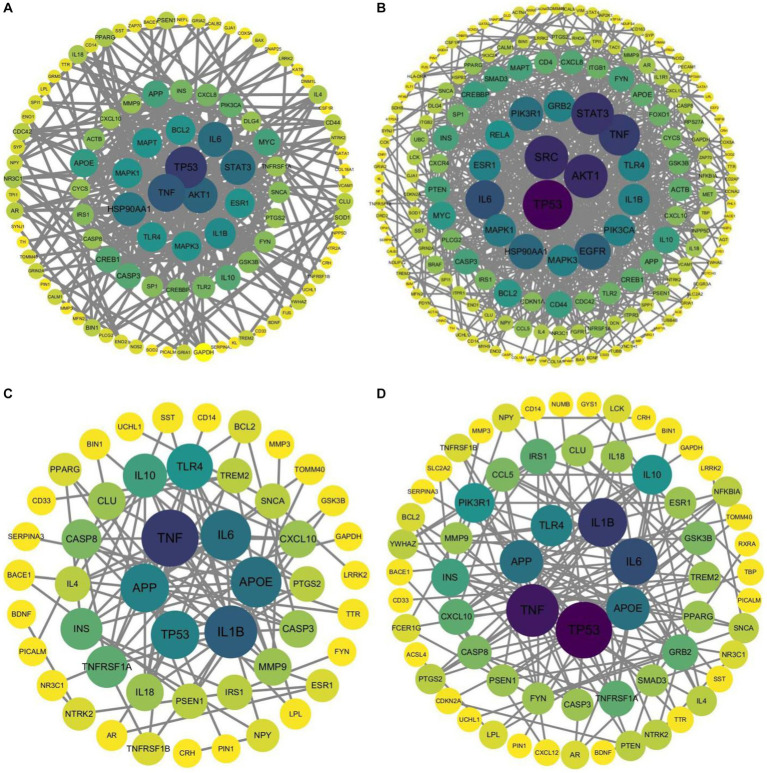
Structural diagram of hub genes identified by PPI analysis, **(A)** AD-RG_DisGeNet, **(B)** AD-RG_GeneCard, **(C)** intersection of all, **(D)** AD-RG_AlzGene.

In addition, PPI analysis was performed on the three gene lists using the STRING database. The PPI analysis results were imported into Cytoscape (version 3.10) to generate a structural diagram of the hub genes. The PPI analysis structural diagram of the hub genes is presented in Figure 4. Furthermore, Table 9 displays the top 10 hub genes generated from the three gene lists.

**Table 9:** Top 10 hub genes identified from three gene lists.

Gene list	Count	Top 10 hub gene
AD-RG_AlzGene	94	TP53, TNF, IL1B, IL6, APOE, APP, TLR4, IL10, PIK3R1, INS
AD-RG_GeneCard	229	TP53, AKT1, SRC, STAT3, TNF, IL6, EGFR, HSP90AA1, PIK3R1, MAPK1
AD-RG_DisGeNet	138	TP53, TNF, AKT1, STAT3, IL6, HSP90AA1, IL1B, ESR1, MAPK1, MAPK3
Intersection of all	63	TP53, TNF, IL1B, IL6, APOE, APP, TLR4, IL10, INS, GRB2

The results in Figure 3 show that cancer-related genes such as TP53 and TNF have higher degree values in the protein interaction network. It may be because this article collected shared genes related to AD-related diseases, and shared genes connect the disease network. This conclusion requires further exploration and verification by experts in the field. Based on this assumption, this paper also draws the interaction network between pathways, see Supplementary Figures S1–S4.

We conducted gene analysis and investigation on the top 10 PPI results generated by the 4 sets of gene lists. TP53, STAT3, EGFR, MAPK1, GRB2, and HSP90AA1 are genes involved in cellular processes. TP53, also known as the tumor suppressor gene p53 (Feng et al., 2011), plays a vital role in maintaining genomic integrity by regulating cell cycle progression and the DNA damage response. It serves as a guardian of the genome and is involved in DNA break repair. TP53 can be targeted by approved drugs (Lagisetty et al., 2022). Additionally, TP53 acts as a transcription factor and influences various aging-related pathways, such as apoptosis, senescence, and insulin/mTOR signaling, which have implications for longevity (Balmorez et al., 2023). STAT3 encodes a transcription factor called Signal Transducer and Activator of Transcription 3 (STAT3). This gene is a key regulator of cellular processes, including cell growth, differentiation, survival, and immune responses. It plays a crucial role in mediating the signaling of cytokines and growth factors. Dysregulation of the JAK2/STAT3 axis, as observed in AD, can lead to cholinergic dysfunction and memory impairment (Chiba et al., 2009). EGFR, or Epidermal Growth Factor Receptor, encodes a receptor protein belonging to the ErbB family of receptor tyrosine kinases. It plays a critical role in regulating cell growth, proliferation, and survival. Choi et al. (2023) demonstrated the potential therapeutic effects of anti-cancer EGFR tyrosine kinase inhibitors (TKIs) on AD pathology. In AD mouse models, EGFR inhibitors have shown promise in attenuating amyloid-beta (Aβ) pathology and improving cognitive function. MAPK1, also known as ERK2, is a member of the mitogen-activated protein kinase (MAPK) family. It is involved in various cellular processes, including cell growth, differentiation, and survival. MAPK1 is a key component of the MAPK signaling pathway, which regulates gene expression and is crucial for neuronal function and plasticity. The MAPK1 gene, also known as Mitogen-Activated Protein Kinase 1 or ERK2 (Extracellular Signal-Regulated Kinase 2), encodes a protein kinase that is a key component of the MAPK signaling pathway. MAPK1 is involved in transmitting signals from the cell surface to the nucleus, regulating various cellular processes, including cell proliferation, differentiation, survival, and apoptosis. Hyperphosphorylation of tau is a key factor in the generation of neurofibrillary tangles (NFTs). MAPK1 and protein kinase C beta (PRKCB) are thought to play a role in hyperphosphorylation, and PRCKB is thought to be involved in hypoxic stress and vascular dysfunction, triggering MAPK phosphorylation pathways (Gerschuetz et al., 2014). The GRB2 (Growth Factor Receptor-Bound Protein 2) gene encodes an adapter protein that plays a critical role in signal transduction pathways. GRB2 is involved in mediating signaling from receptor tyrosine kinases, such as the epidermal growth factor receptor (EGFR), to downstream signaling molecules. It acts as a bridge between activated receptors and intracellular signaling proteins, facilitating the transmission of signals that regulate various cellular processes. Grb2 and p38α MAPK are important for atherosclerosis and neointima formation (Proctor, 2008). Majumder et al. (2017) unravel a the unique role of Grb2 in protecting the cytoskeletal architecture in AD-like conditions. The HSP90AA1 gene encodes a heat shock protein called HSP90 alpha, also known as HSP90AA1 or HSPC1. HSP90 is a highly conserved molecular chaperone that plays a crucial role in protein folding, stability, and degradation. It is involved in various cellular processes, including signal transduction, cell cycle regulation, and protein quality control. Qian et al. (2022) identified HSP90AA1 as a reliable immune hub gene in patients with mild cognitive impairment (MCI) and consistent changes in AD. The expression level of HSP90AA1 was negatively correlated with alpha- and beta-secretase activity, suggesting its involvement in AD pathology.

APOE and APP are widely recognized as prominent genetic factors associated with AD. Despite the lack of success in translating anti-amyloid therapeutic strategies into clinically effective treatments, it has been suggested that APP and Aβ42 may not be the sole contributors to the AD disease cascade. Nevertheless, the amyloid hypothesis continues to be regarded as a significant mechanism underlying the pathophysiology of AD. In addition to the noteworthy correlation between APP and PSEN mutations in familial AD cases, APOE4 has consistently emerged as the most robust risk factor for late-onset AD to date (Li et al., 2017).

IL1B, IL6, TNF, TLR4, TNF, and SRC are genes associated with inflammation and immune responses that have been linked to AD. IL1B has been found to play a promoting role in neuroinflammation by enhancing the expression of leukocyte chemotactic chemokines, cell surface adhesion molecules, cyclooxygenases, and MMPs within the brain parenchyma (Knopman et al., 2021). Additionally, IL1B may contribute to the peripheral systemic host immune response triggered by periodontitis, leading to central nervous system (CNS) dysfunction in AD (Jin et al., 2021). In the immune system category, cytokines such as IL6 and TNF alpha play crucial roles in regulating inflammatory pathways, including neuroinflammation in AD. Aß plaques have been shown to increase the levels of these proinflammatory cytokines, resulting in a cycle of inflammation and plaque accumulation (Lin et al., 2021). On the other hand, interleukin-10 (IL-10) acts as an important anti-inflammatory cytokine with potential anti-atherogenic properties. Toll-like receptors (TLRs) are pattern recognition receptors that play a central role in regulating the host’s protective adaptive immune response. Among the TLR family members, TLR4 is widely expressed in neural cells, including microglia, neurons, astrocytes, and endothelial cells (Lin et al., 2021). Tumor necrosis factor (TNF) is a small protein mainly secreted by macrophages and is involved in various cellular processes, including activating the NF-кB signaling pathway, promoting cell death, and regulating immune function (Haeberlein et al., 2022). TNF-mediated neuroinflammation has been associated with the necroptosis of hippocampal neurons in AD. The SRC gene encodes Src kinase, a non-receptor tyrosine kinase involved in regulating cell growth, differentiation, adhesion, migration, and survival. SRC-1, a key coactivator of SRC, is abundant in the hippocampus and has been implicated in cognition. It is also related to major risk factors for AD, such as estrogen decline and aging (Wu et al., 2020).

The genes PIK3R1, INS, and AKT1 are known to be involved in metabolic processes. PIK3R1, also known as p85a, functions as a regulatory subunit of phosphoinositide 3-kinases (PI3Ks). It plays a crucial role in stabilizing and inhibiting the catalytic activity of p110 and acts as an adaptor to interact with insulin receptor substrate (IRS) proteins and growth factor receptors. Mutations or altered expression of PIK3R1 can modulate the activity of PI3K, leading to significant metabolic outcomes (Tsay and Wang, 2023). The INS gene is responsible for encoding insulin, a hormone that is secreted by the pancreas. Insulin plays a vital role in regulating blood glucose levels and energy metabolism. Its primary function is to facilitate the uptake and utilization of glucose. Dysregulation of glucose metabolism has been implicated in the development of AD, with genetic variations in INS and PPARA, particularly among Northern Europeans, potentially contributing to this dysregulation (Koelsch et al., 2012). The AKT1 gene encodes a protein kinase called AKT kinase. AKT1 is a key molecule involved in cellular signaling pathways and is responsible for regulating various biological processes, including cell survival, proliferation, growth, and metabolism. Evidence suggests that the AKT1 protein may be associated with an increased risk of AD, especially among patients with type 2 diabetes (Liu et al., 2015).

### 5.4 Tissue-specific analysis

Import GTEx data to allow validation of tissue passes for reference gene lists. We downloaded the latest RNA-Seq TPM data (version 8) from the GTEx Portal website, which contains nearly 1,000 human samples from 54 non-tissue injury sites. To implement gene annotation based on GENCODE (v44), we deleted genes less than 1 kb in length. We deleted the expression data of four tissues with sample sizes less than 15: renal medulla, ectocervix, fallopian tube, and endocervix.

First, gene co-expression patterns were used to assess the functional relevance of susceptibility genes. Use the bicor function in the WGCNA package to calculate the correlation coefficient between two genes, and set the correlation coefficient threshold to count the gene amount. If the correlation coefficient between two genes is greater than 0.8, there is connectivity between genes. We calculated the correlation coefficient and gene connectivity of the expression data of the 4 reference gene lists AD-RG_AlzGene, AD-RG_GeneCard, AD-RG_DisGeNet, and ALL on 50 tissues in GTEx. Then, for each gene list, 10,000 random gene lists with the same number of genes were generated through resampling, and the average correlation coefficient and average gene connect amount of the random gene list were calculated. Calculation results are stored in Supplementary Tables S1–S4.

Then, the specific organization of the reference gene is found based on the Z-score normalized difference. For the four reference gene lists, calculate the Z-score standardized values of the correlation coefficients and gene connect between the reference gene list and the random gene list respectively, and then calculate the Z-score difference between the reference gene list and the random gene list to find the largest difference of 10 organizations.

**Figure 5:**
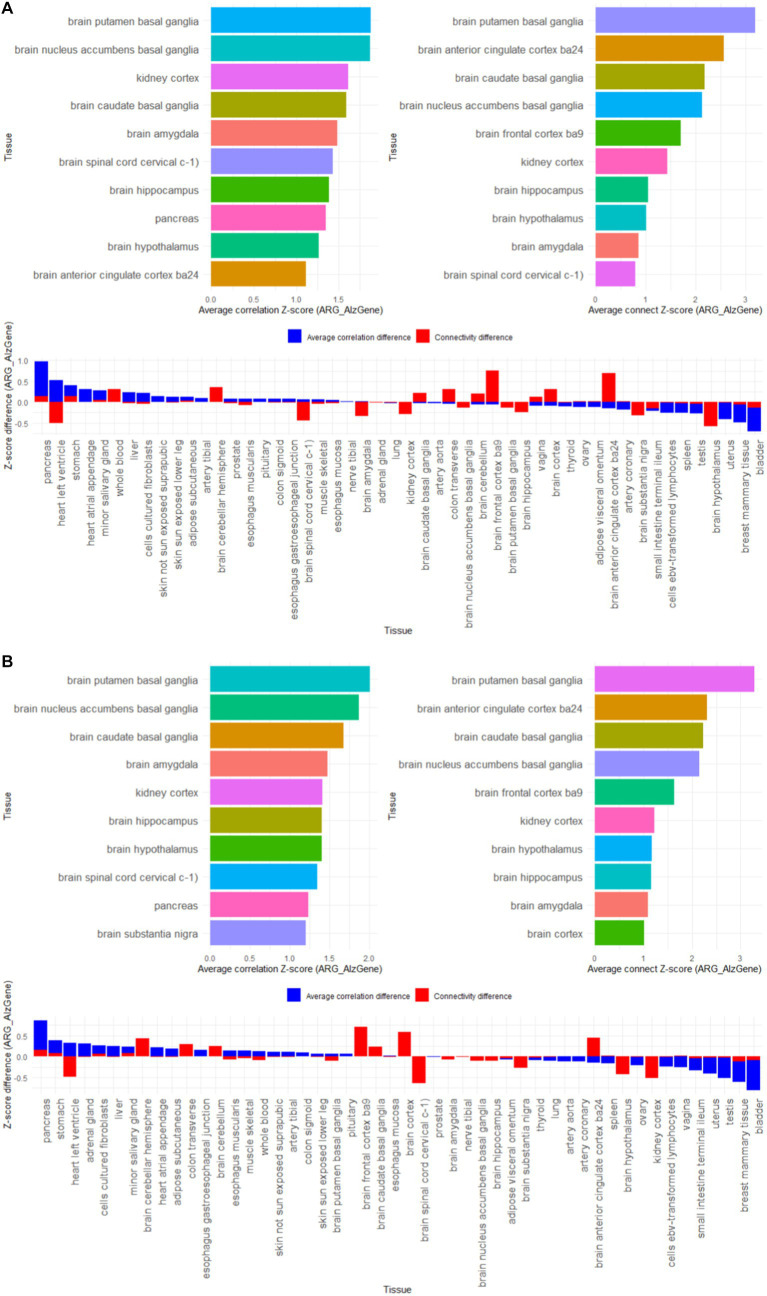

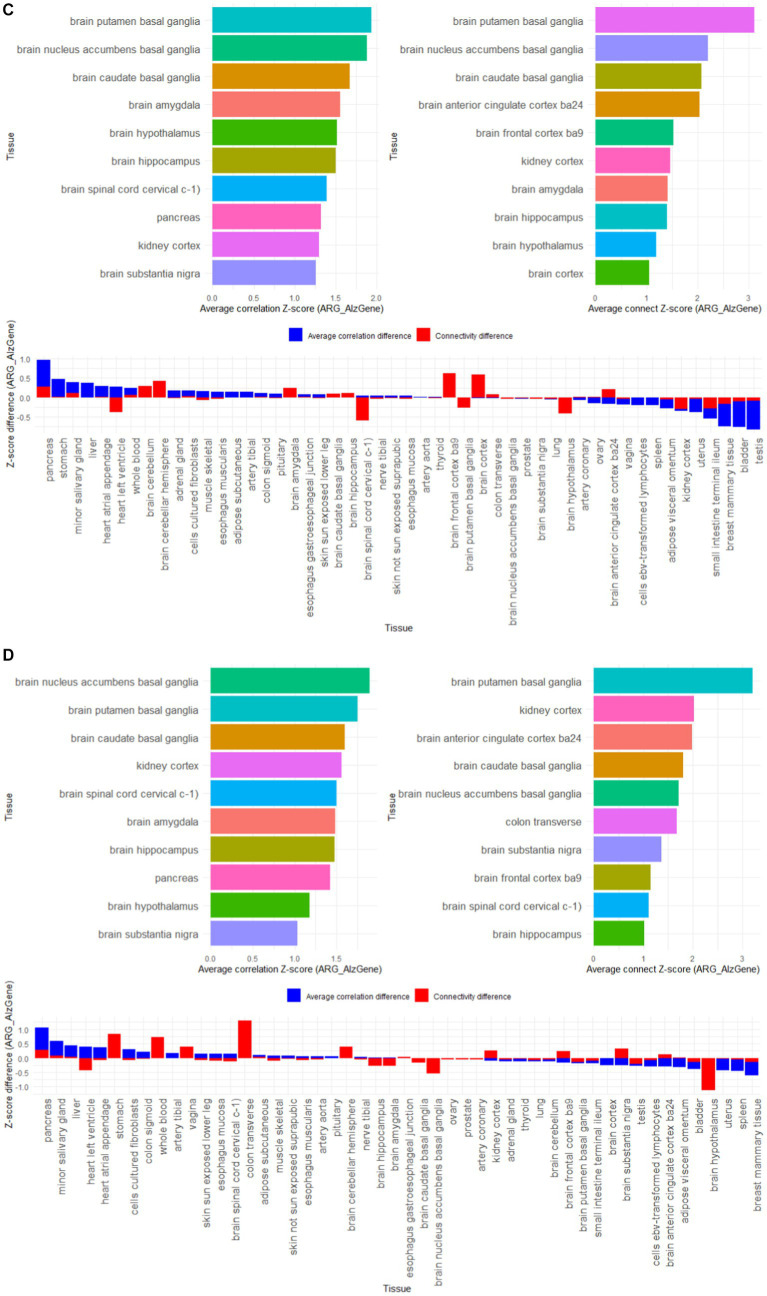
Z-score results of tissue-specific analysis of AD associated hub gene list in GTEx database, with top 10 tissues as association coefficient, top 10 tissues as connectivity, and Z-score differences among different tissues **(A)** AD-RG_AlzGene, **(B)** AD-RG_GeneCard, **(C)** AD-RG_DisGeNet, **(D)** intersection of all.

The results in Figure 5 show that among the 50 tissues, the 4 gene lists we constructed showed high specificity for brain tissues, such as putamen basal ganglia, anterior cingulate cortex ba24, caudate basal ganglia, nucleus accumbens basal ganglia, frontal cortex ba9, hypothalamus, hippocampus, amygdala, cortex, substantia nigra. It is worth noting that the kidney cortex tissue also showed higher characteristics, which coincides with the view that “AD is also called type 3 diabetes.”

## 6 Conclusion

This article reviews recent research efforts in the identification of candidate biomarkers for AD and categorizes them into three main groups. Firstly, it discusses the use of conventional algorithms for AD biomarker identification, including GWAS, differential analysis, WGCNA, machine learning, and deep learning. In the field of bioinformatics, there is still room for improvement in the application of advanced algorithms such as machine learning and deep learning. Secondly, it explores AD biomarkers associated with biological processes such as mitochondrial dysfunction, neuroinflammation, immune dysregulation, aging, metabolism, and apoptosis. Understanding these biological processes is crucial for identifying relevant AD biomarkers. The third category involves the identification of biomarkers shared with co-occurring diseases, including psychiatric disorders, metabolic diseases, inflammatory diseases, viral infections, and cancer. Exploring the overlap between AD and other related diseases can provide valuable insights into common biomarkers and underlying mechanisms. Additionally, this article performs a statistical analysis of key genes mentioned in the research literature and identifies the intersection with AD-related gene sets from databases such as AlzGen and GeneCard. For overlapping genes, enrichment analysis is conducted, and PPI networks are utilized to identify central genes among the overlapping genes. Overall, this article provides a comprehensive overview of recent advances in AD biomarker identification, highlighting the use of various algorithms, the exploration of relevant biological processes, and the investigation of shared biomarkers with co-occurring diseases.

In the future, we aspire to precisely identify candidate biomarkers for AD, enabling early-stage diagnosis and prevention, and offering early intervention and treatment opportunities for patients. By delving into the biological processes associated with AD, such as mitochondrial dysfunction, neuroinflammation, immune dysregulation, and others, we can discover new therapeutic targets and strategies, empowering precision medicine with more effective tools.

## Author contributions

ZZ: Writing – original draft, Writing – review & editing. XL: Supervision, Writing – original draft, Writing – review & editing. SZ: Resources, Writing – review & editing. ZS: Funding acquisition, Writing – review & editing. KL: Funding acquisition, Writing – review & editing. WY: Supervision, Writing – review & editing.

## Footnotes

^1^https://www.who.int/data/gho/publications/world-health-statistics

^2^https://www.alz.org/media/Documents/alzheimers-facts-and-figures.pdf

^3^https://www.cdc.gov/aging/aginginfo/alzheimers.htm
