# Supplementary figures and images for: A review and analysis of key biomarkers in Alzheimer’s disease
Source: Front Neurosci. 2024 Feb 20;18:1358998. doi: 10.3389/fnins.2024.1358998 (PMC10912539; doi:10.3389/fnins.2024.1358998)

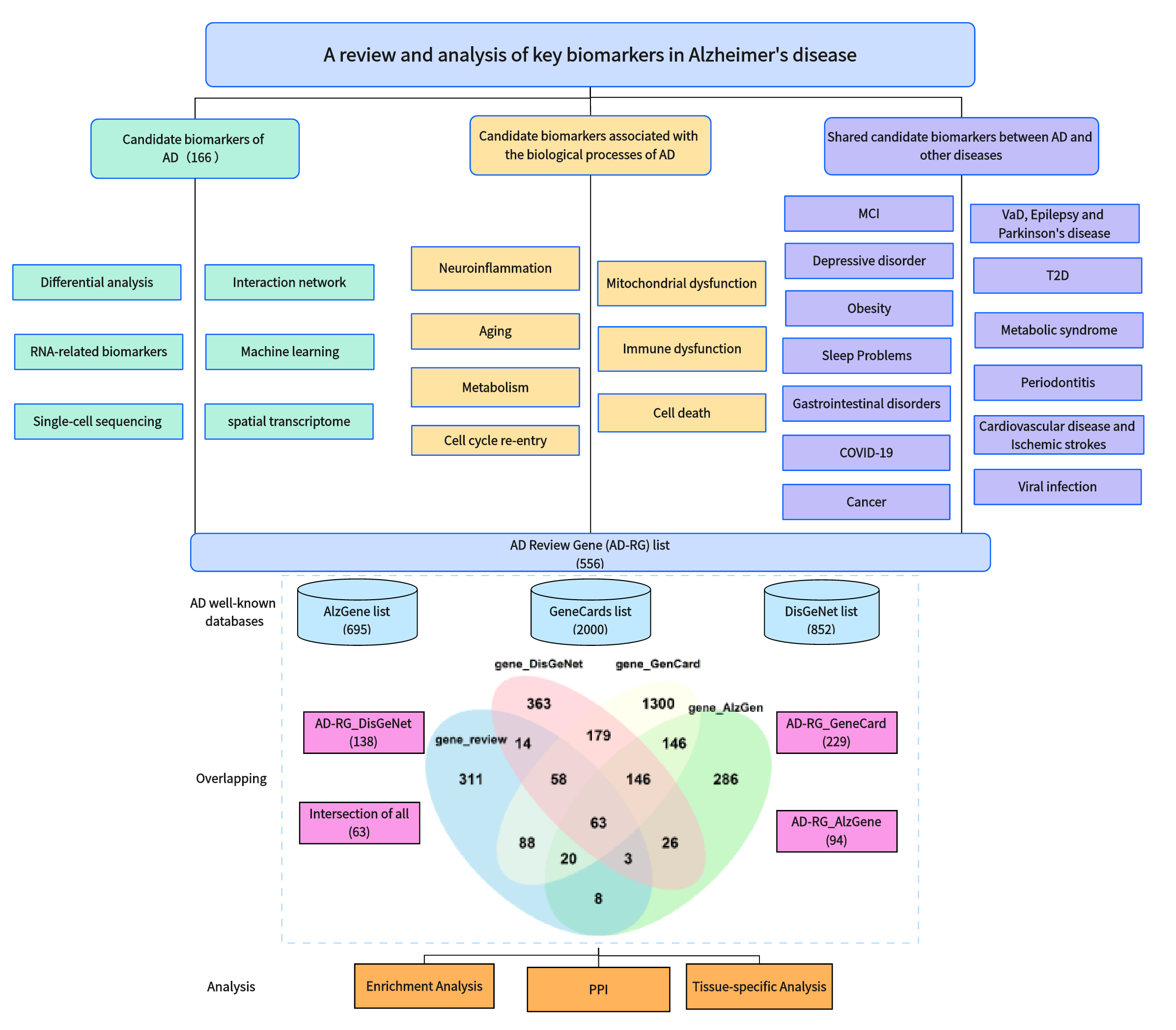

Supplement: Supplementary file 1 [file Data_Sheet_1.ZIP › Figure 1 Article workflow diagram.png]

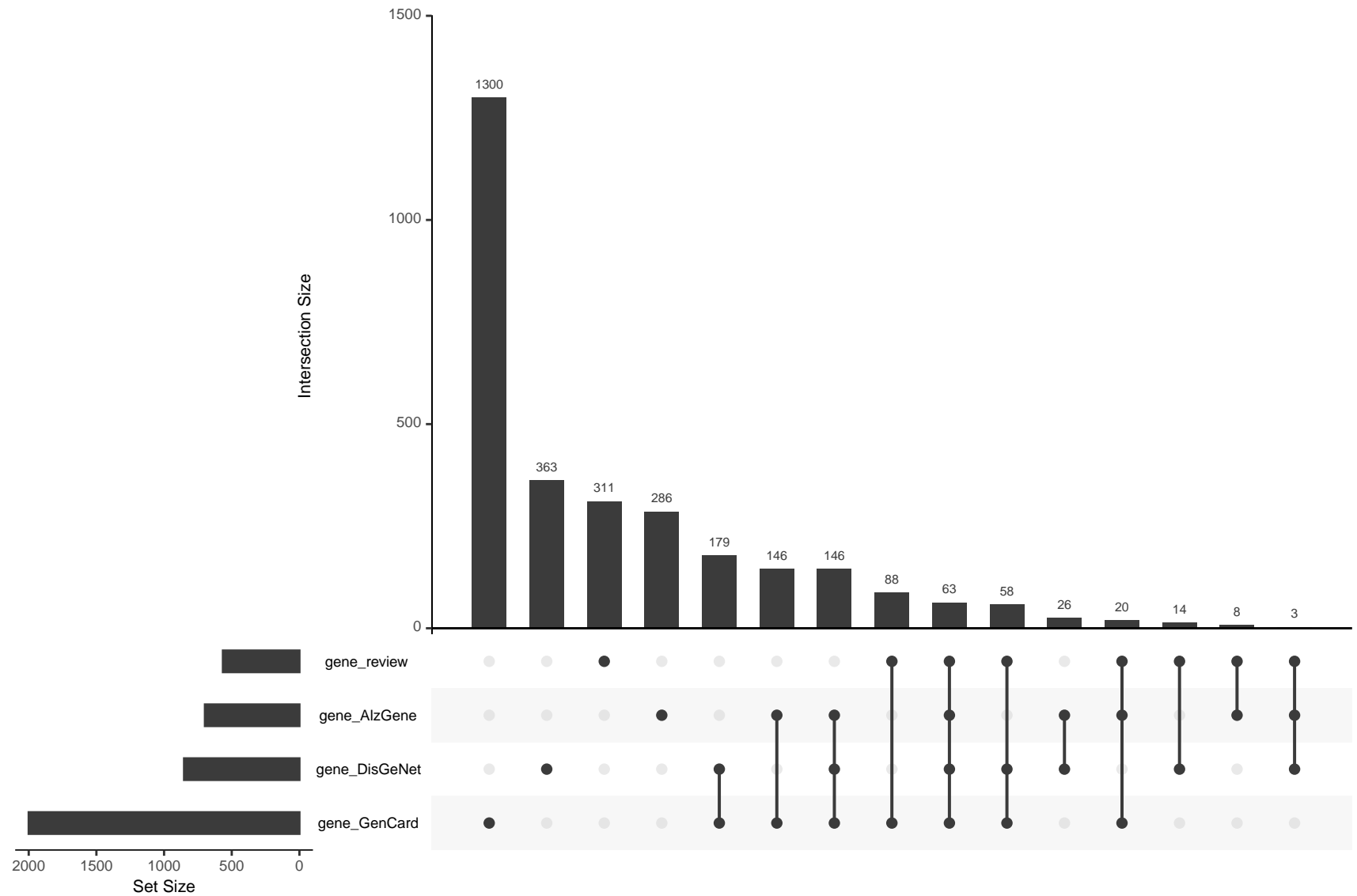

Supplement: Supplementary file 1 [file Data_Sheet_1.ZIP › Figure 2 Upset plot result.pdf]

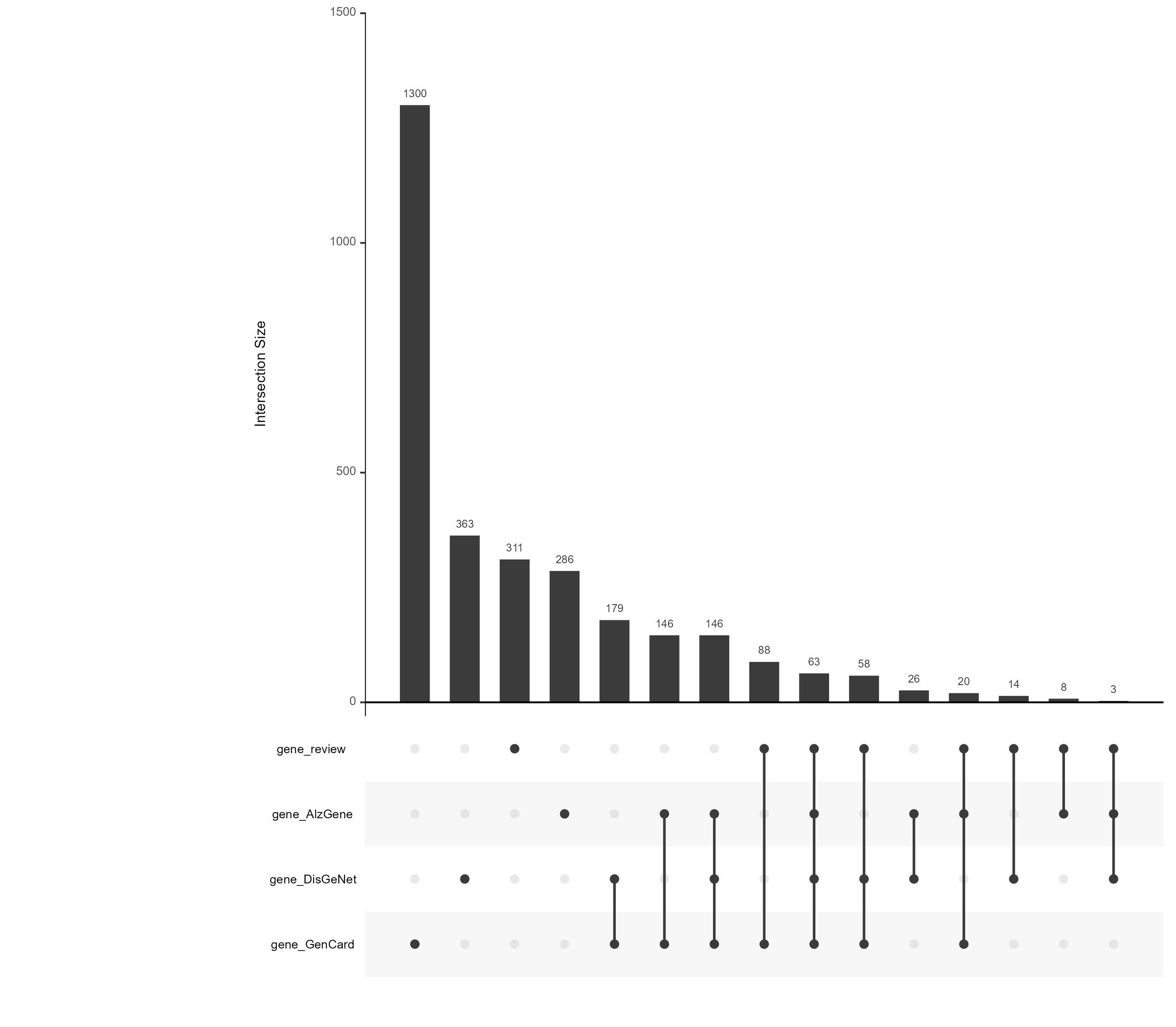

Supplement: Supplementary file 1 [file Data_Sheet_1.ZIP › Figure 2 Upset plot result.pdf.png]

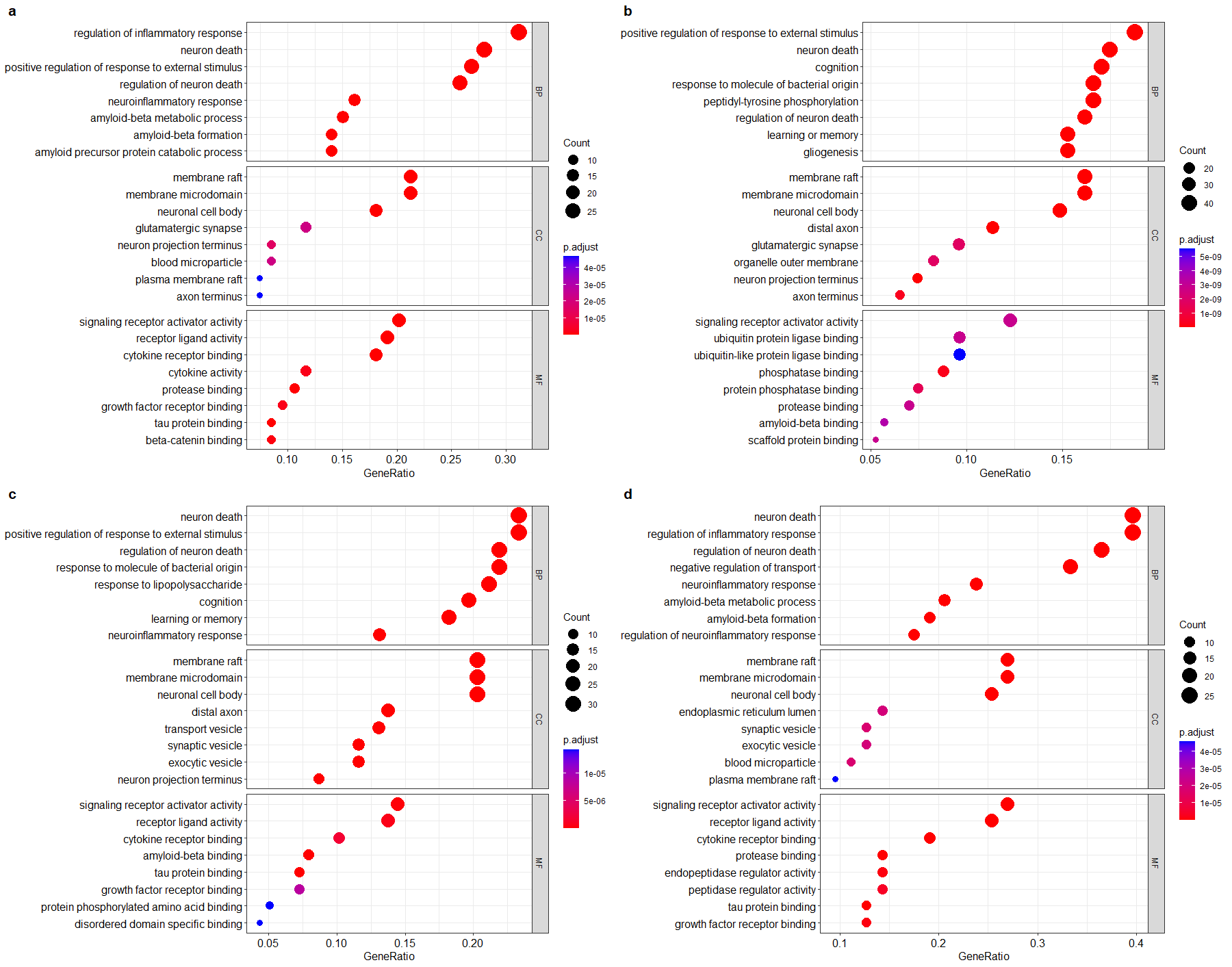

Supplement: Supplementary file 1 [file Data_Sheet_1.ZIP › Figure 3 GO Enrichment analysis.png]

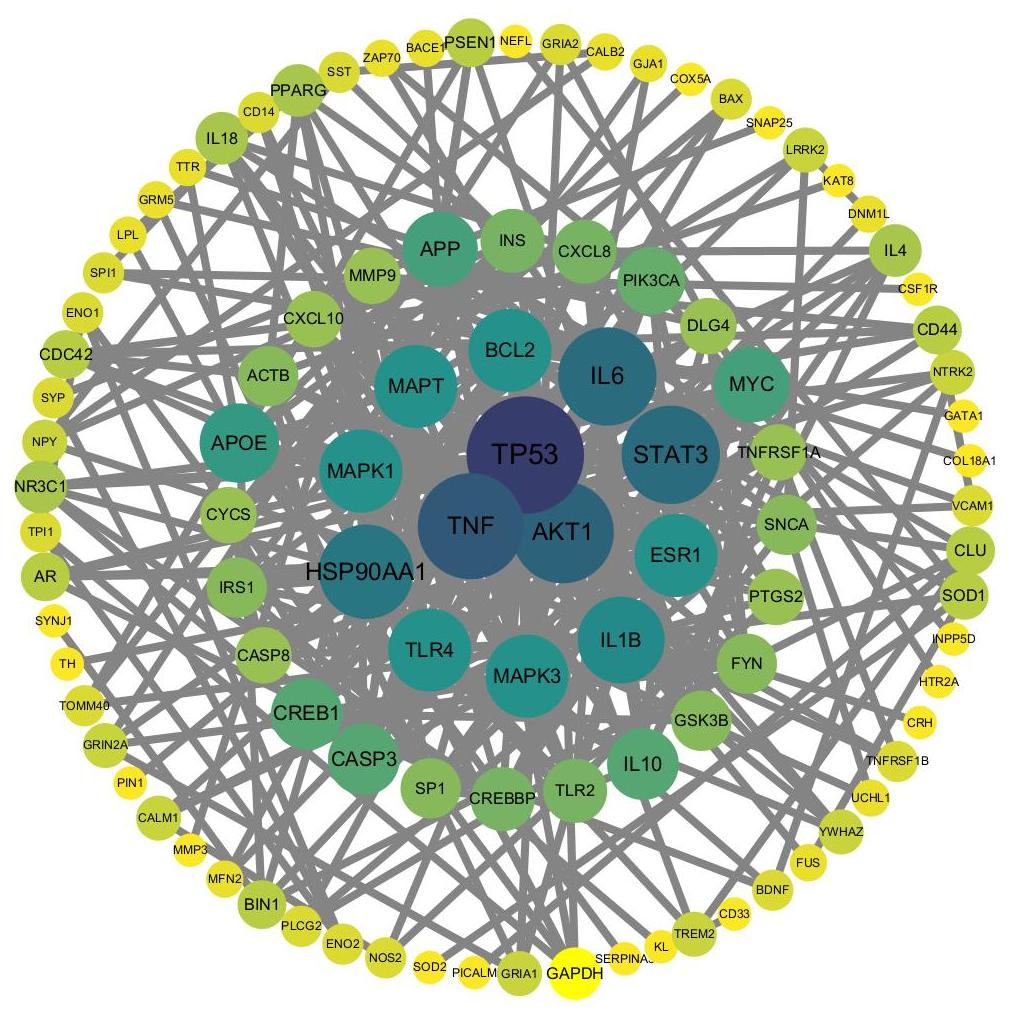

Supplement: Supplementary file 1 [file Data_Sheet_1.ZIP › Figure 4(A) AD-RG_DisGeNet structural Diagram of Hub Genes.jpeg]

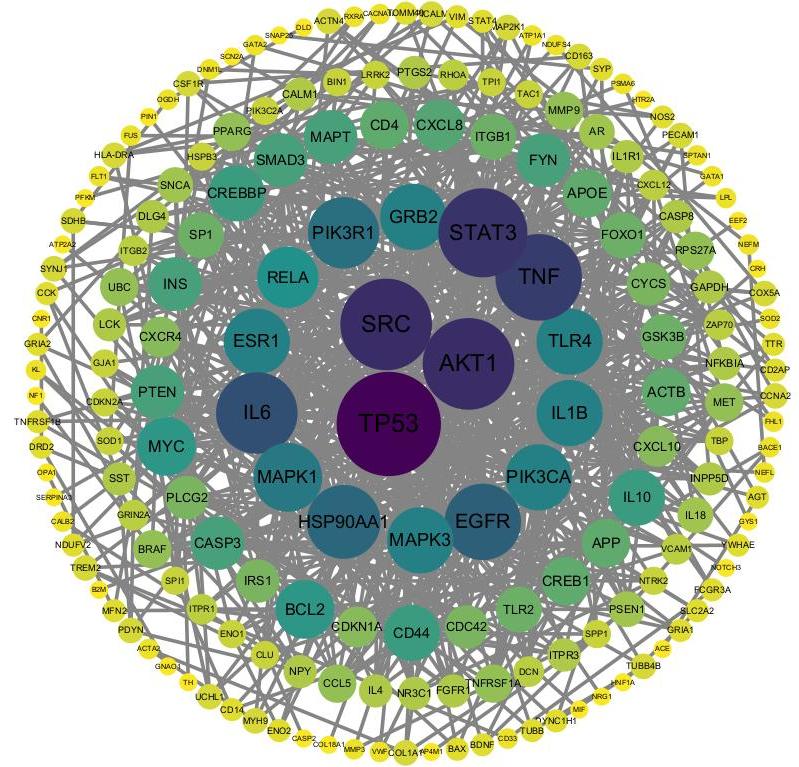

Supplement: Supplementary file 1 [file Data_Sheet_1.ZIP › Figure 4(B) AD-RG_GeneCard structural Diagram of Hub Genes.jpeg]

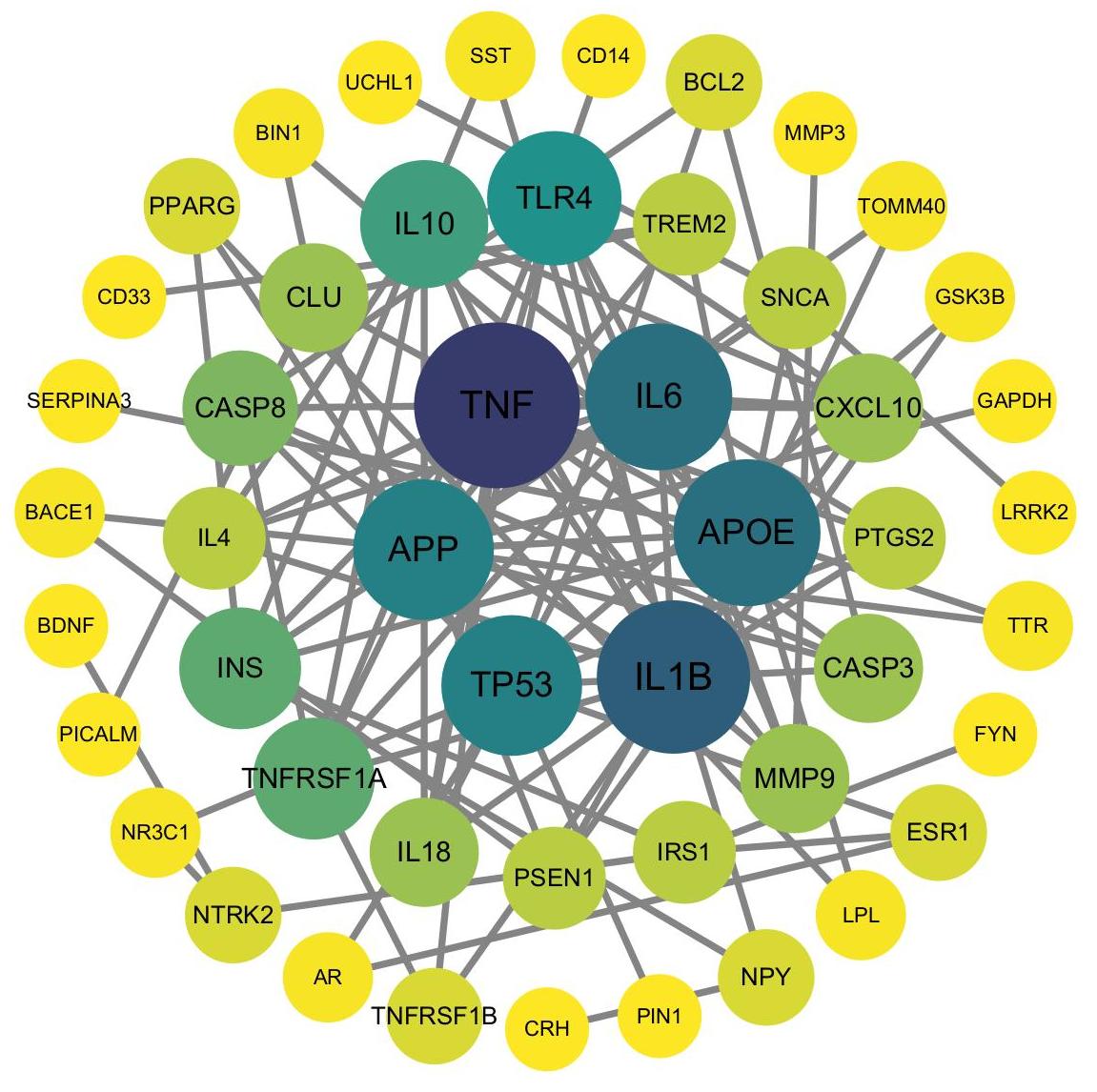

Supplement: Supplementary file 1 [file Data_Sheet_1.ZIP › Figure 4(C) ALL_interaction structural Diagram of Hub Genes.jpeg]

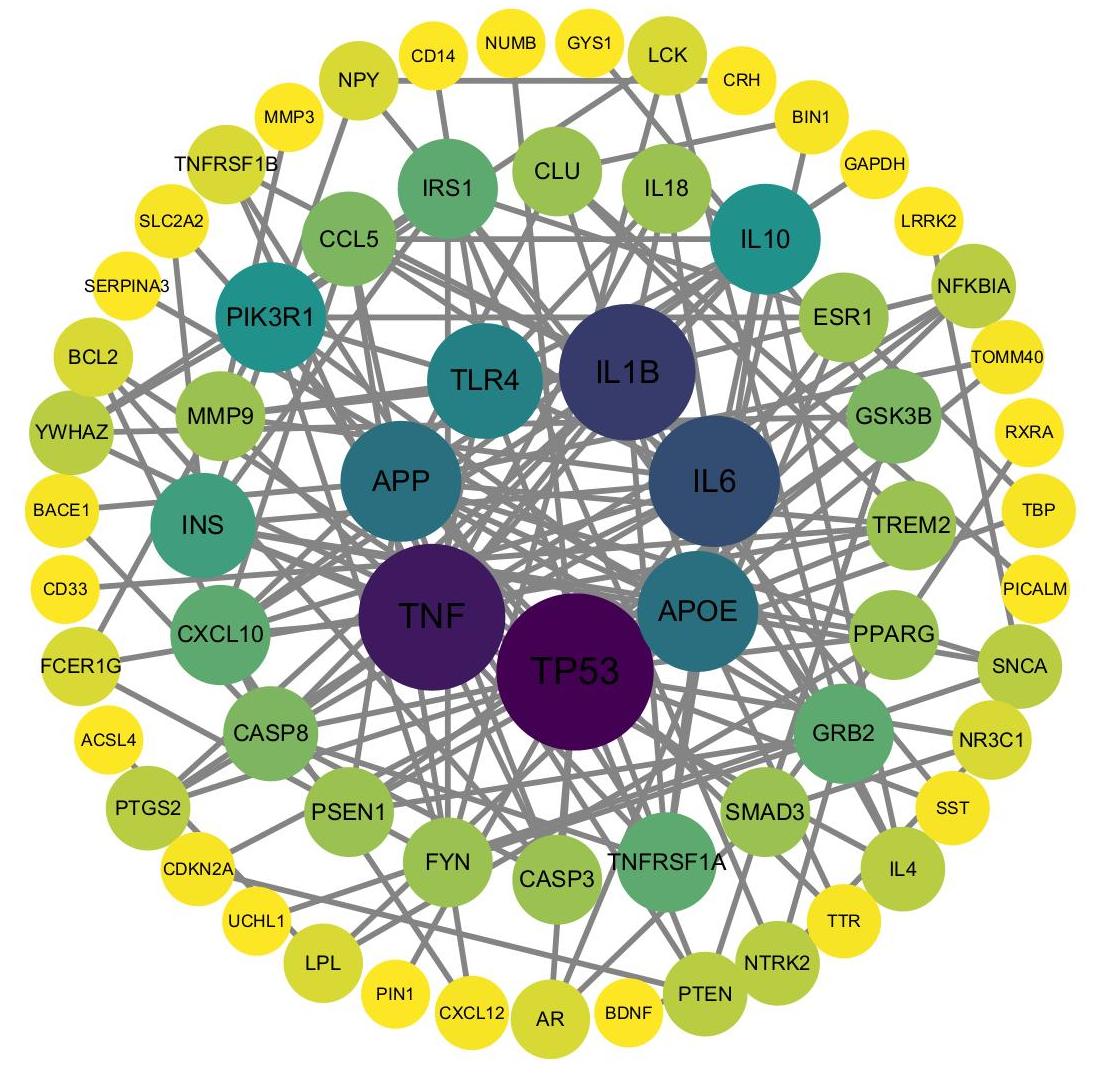

Supplement: Supplementary file 1 [file Data_Sheet_1.ZIP › Figure 4(D) AD-RG_AlzGene structural Diagram of Hub Genes.jpeg]

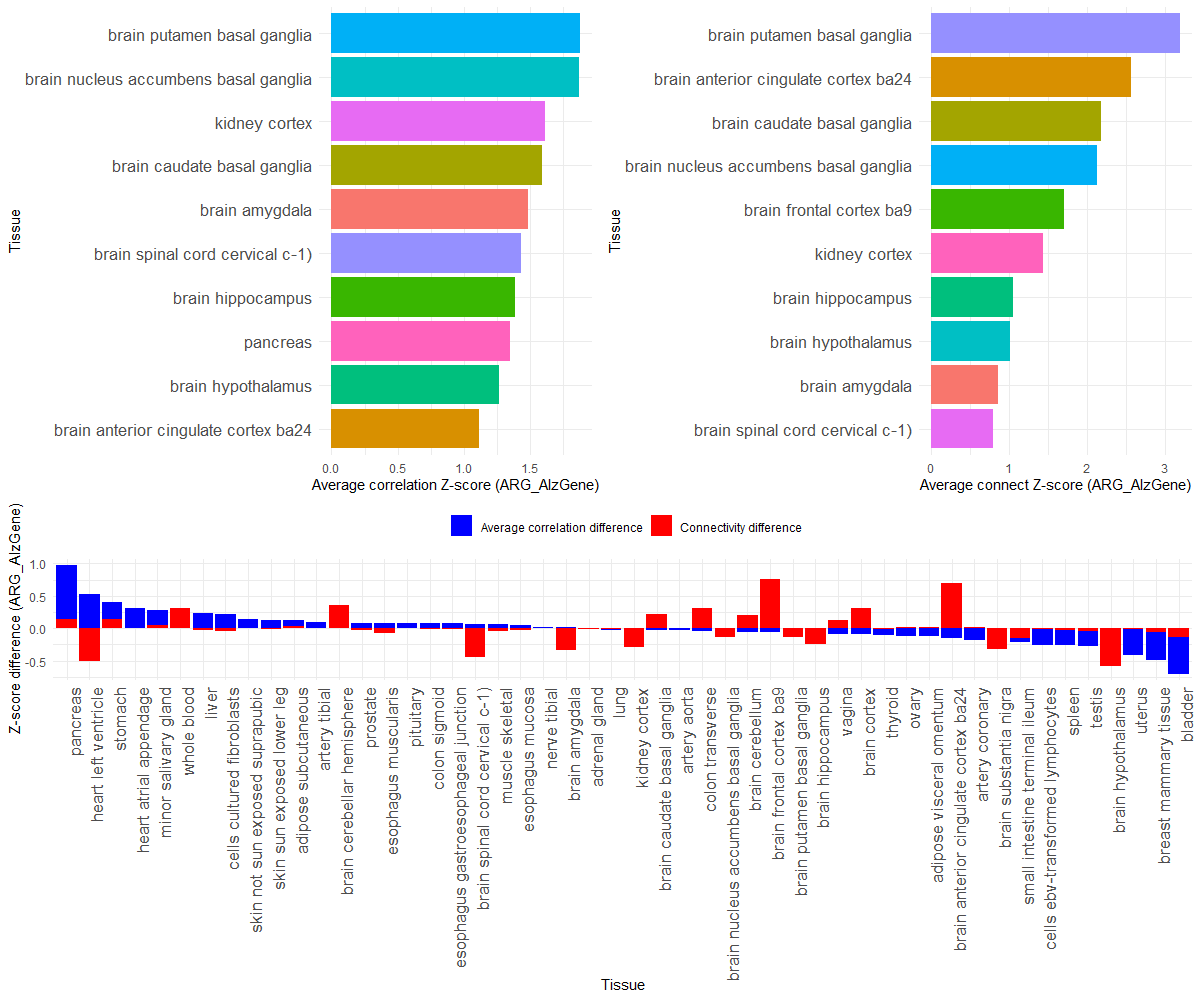

Supplement: Supplementary file 1 [file Data_Sheet_1.ZIP › Figure 5(A) ARG_AlzGene_combined_plot3.png]

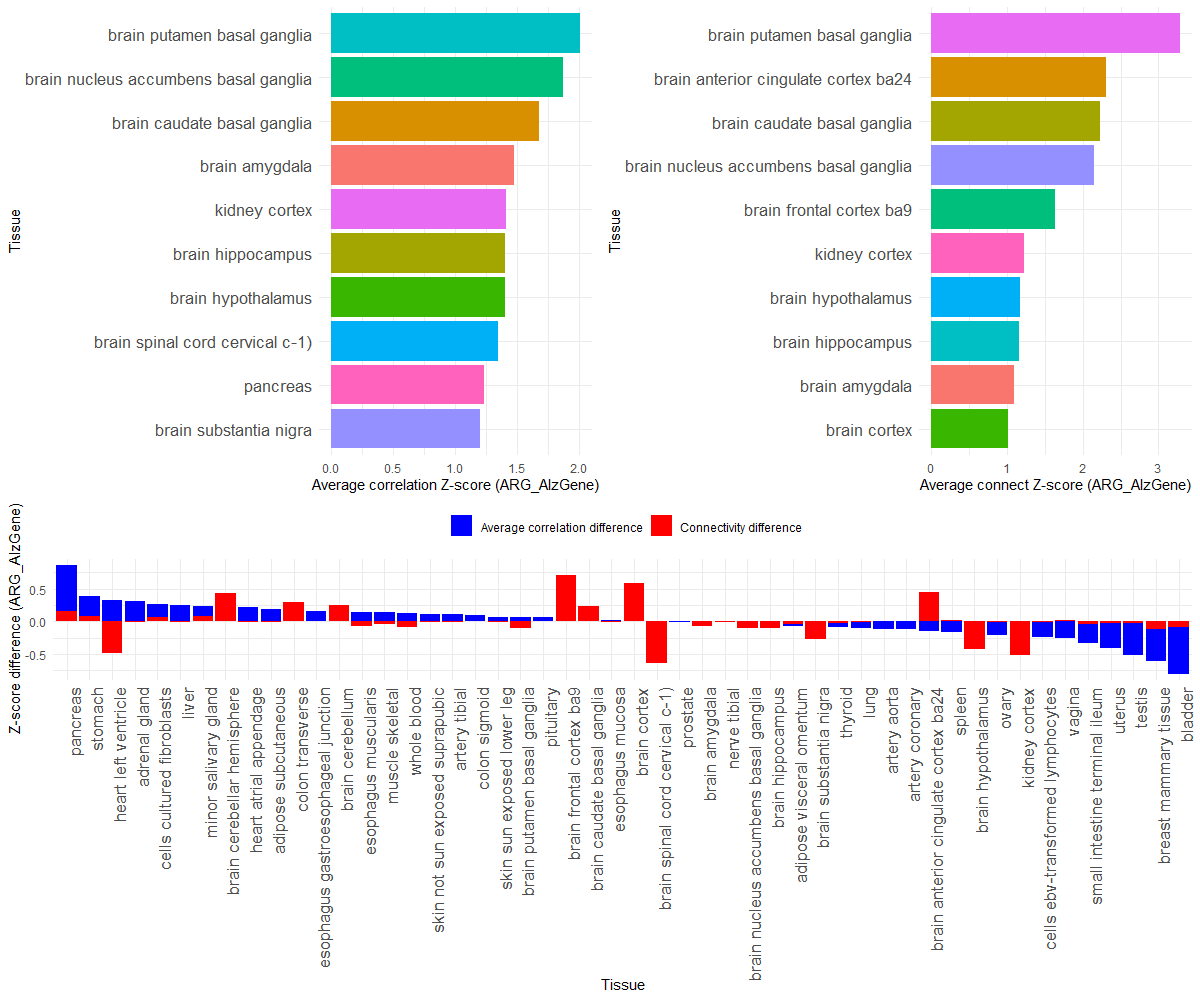

Supplement: Supplementary file 1 [file Data_Sheet_1.ZIP › Figure 5(B) ARG_GenCard_combined_plot3.png]

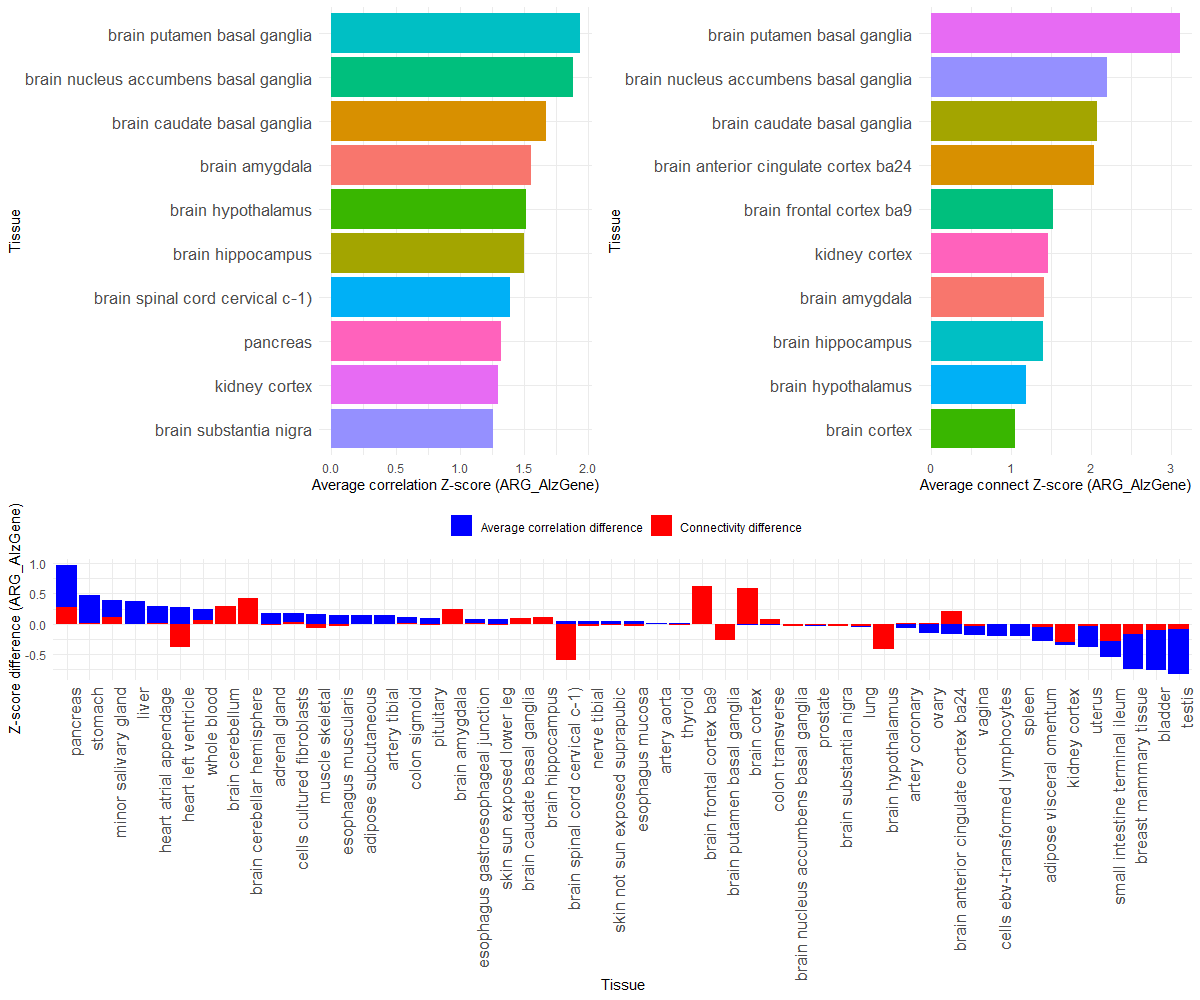

Supplement: Supplementary file 1 [file Data_Sheet_1.ZIP › Figure 5(C) ARG_DisGeNet_combined_plot3.png]

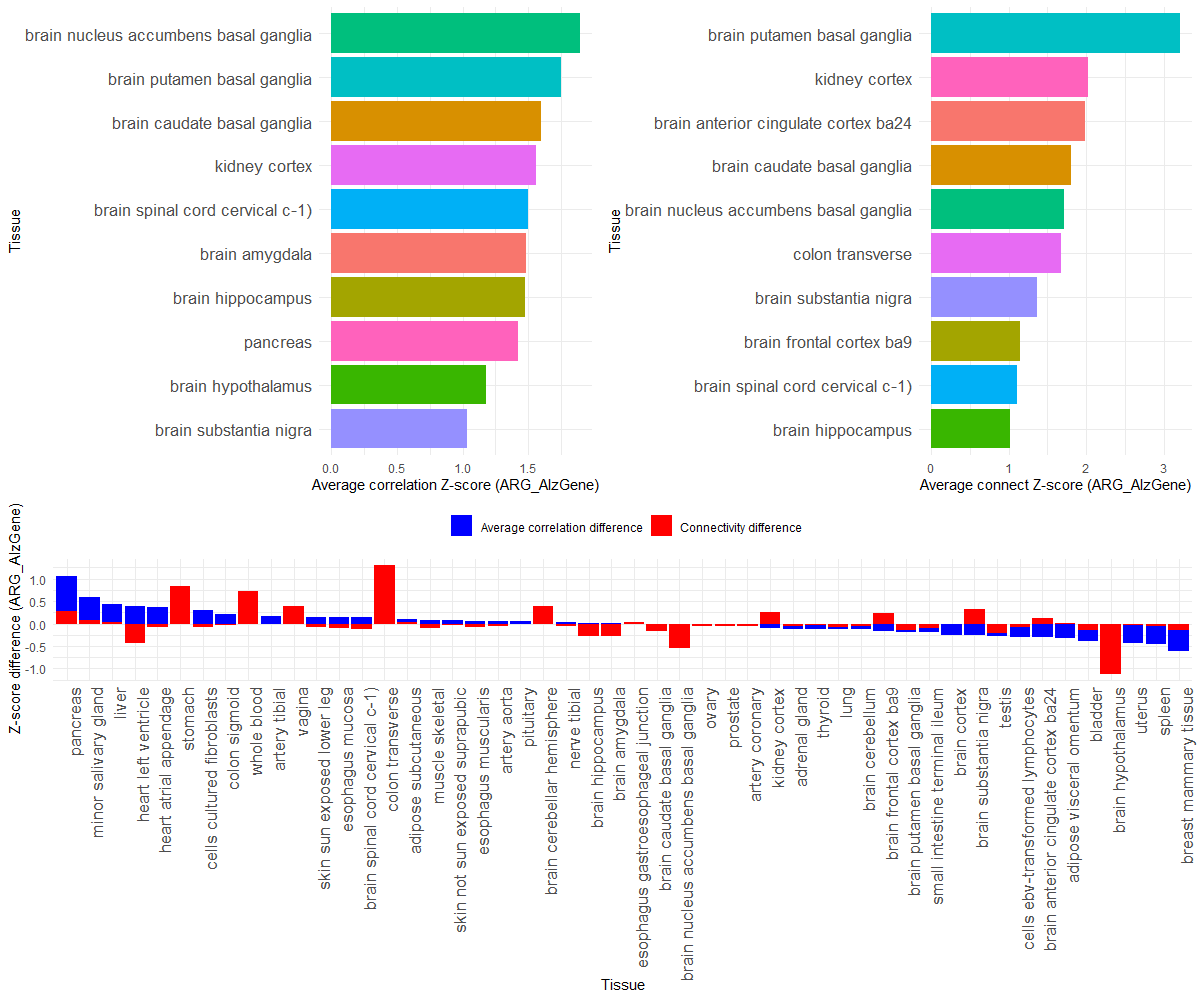

Supplement: Supplementary file 1 [file Data_Sheet_1.ZIP › Figure 5(D) ALL_interaction_combined_plot3.png]
